# Analyzing greedy vaccine allocation algorithms for metapopulation disease models

**DOI:** 10.1371/journal.pcbi.1012539

**Published:** 2025-07-21

**Authors:** Jeffrey Keithley, Akash Choudhuri, Bijaya Adhikari, Sriram V. Pemmaraju

**Affiliations:** Department of Computer Science, University of Iowa, Iowa City, Iowa, United States of America; The University of Melbourne Faculty of Science, AUSTRALIA

## Abstract

As observed in the case of COVID-19, effective vaccines for an emerging pandemic tend to be in limited supply initially and must be allocated strategically. The allocation of vaccines can be modeled as a discrete optimization problem that prior research has shown to be computationally difficult (i.e., NP-hard) to solve even approximately. Using a combination of theoretical and experimental results, we show that this hardness result may be circumvented. We present our results in the context of a metapopulation model, which views a population as composed of geographically dispersed heterogeneous subpopulations, with arbitrary travel patterns between them. In this setting, vaccine bundles are allocated at a subpopulation level, and so the vaccine allocation problem can be formulated as a problem of maximizing an integer lattice function $g:{\mathbb{Z}}_{+}^{K}\to \mathbb{R}$ subject to a budget constraint $\|x{\|}_{1}\leq D$. We consider a variety of simple, well-known greedy algorithms for this problem and show the effectiveness of these algorithms for three problem instances at different scales: New Hampshire (10 counties, population 1.4 million), Iowa (99 counties, population 3.2 million), and Texas (254 counties, population 30.03 million). We provide a theoretical explanation for this effectiveness by showing that the approximation factor (a measure of how well the algorithmic output for a problem instance compares to its theoretical optimum) of these algorithms depends on the *submodularity ratio* of the objective function *g*. The submodularity ratio of a function is a measure of how distant *g* is from being submodular; here submodularity refers to the very useful “diminishing returns” property of set and lattice functions, i.e., the property that as the function inputs are increased the function value increases, but not by as much.

## Author summary

Strategic and timely allocation of vaccines is crucial in combating epidemic outbreaks. Developing strategies to allocate vaccines over subpopulations rather than to individuals leads to policy recommendations that are more feasible in practice. Despite this, vaccine allocation over subpopulations has only received limited research interest, and the associated computational challenges are relatively unknown. To address this gap, we study vaccine allocation problems over geographically distinct subpopulations in this paper. We formulate our problems to reduce either **i)** the total infections or **ii)** the sum of peak infections over meta-population disease models. We first demonstrate that these problems are computationally challenging even to approximate and then show that a family of simple, well-known greedy algorithms exhibit provable guarantees. We conduct realistic experiments on state-level mobility graphs derived from real-world data in three states of distinct population levels: New Hampshire, Iowa, and Texas. Our results show that the greedy algorithms we consider are **i)** scalable and **ii)** outperform both state-of-the-art and natural baselines in a majority of settings.

## Introduction

In the early stages of a pandemic like COVID-19, the demand for vaccinations far exceeds supply [1,2] and it is critical to strategically allocate vaccines [3,4]. The vaccine allocation problem can be modeled in a variety of ways, including as discrete optimization problems [5–9].

However, all of these problems are computationally hard, even to solve approximately (see [10], for a specific example). Despite these obstacles, we need to be able to solve vaccine allocation problems at scale and have confidence that the obtained solutions are close to being optimal. In this paper, we take steps towards this goal.

We consider the metapopulation-network model for disease-spread [11,12], which allows for heterogeneity among geographically distinct subpopulations and arbitrary travel patterns between them. Vaccine allocation within this model consists of allocating some number of bundles of vaccines to each subpopulation while satisfying an overall budget constraint. The resulting family of problems, which we call the *Metapopulation Vaccine Allocation (MVA)* problems, can be formalized as maximizing an objective function (e.g., number of cases averted) defined over an integer lattice domain subject to a budget constraint. Here, the integer lattice refers to the set of all possible vaccine allocations, where each allocation assigns an integer number of vaccines to each subpopulation. Not surprisingly, we show specific problems obtained via realistic instantiations of the metapopulation-network model and objective function in MVA are not just NP-hard (i.e., computationally intractable in general), but even hard to approximate. We show these hardness results for two instantiations, which we call MaxCasesAverted and MaxPeaksReduced, of MVA over SEIR (**S**usceptible- **E**xposed- **I**nfected- **R**ecovered) metapopulation models [11,12]. These hardness of approximation results imply that worst-case approximation guarantees are not attainable for natural instantiations of MVA. However, for a family of simple, well-known greedy algorithms, we show positive theoretical and experimental results for both MaxCasesAverted and MaxPeaksReduced. These simple and natural greedy algorithms lend themselves to the machinery from submodular function optimization for in-depth analysis. There is a rich literature of methods for submodular set function optimization [13–18] that has subsequently been extended to submodular optimization over the integer lattice [19–22]. In general, submodularity is a valuable property for a function being maximized, as it imposes structured behavior when elements are added to its input, enabling us to bound how far the value of a greedy solution is from the optimal value. Furthermore, in the last few years, researchers have attempted to extend some of the aforementioned results for submodular set and lattice function optimization to functions that are not submodular, by using the notion of *submodularity ratio* of a function, which is a measure of how distant that function is from being submodular [23–25]. All of this literature is foundational to our approach to analyzing vaccine allocation algorithms in a metapopulation model setting [11,12].

In our main theoretical result, we show that simple greedy algorithms provide worst-case approximation guarantees for MaxCasesAverted and MaxPeaksReduced that become better as the submodularity ratio of their objective functions approaches 1. The submodularity ratio [23–26] of a set or lattice function is a measure (between 0 and 1) of how close the function is to being submodular, with values closer to 1 corresponding to functions that are closer to being submodular. We complement this theoretical result with experimental results indicating that the objective functions for MaxCasesAverted and MaxPeaksReduced might have relatively high submodularity ratios.

We then experimentally evaluate the performance of greedy vaccine allocation algorithms at three scales; we use New Hampshire (10 counties, population 1.4 million) for our small scale experiments, Iowa (99 counties, population 3.2 million) for our medium scale experiments, and Texas (254 counties, population 30.03 million) for our large scale experiments. We compare the performance of the greedy methods with a set of trivial baselines, such as allocating vaccines according to population sizes. We also compare against a randomized algorithm called Pareto Optimization for Subset Selection (POMS) [24]. POMS works by expanding a random pareto-optimal frontier (i.e., interpreting solution size as a second objective function and finding solutions which balance the quality of both objective functions), and was designed to compete against greedy algorithms for small scale problems. We show the greedy algorithms we consider outperform POMS for our experimental settings, while scaling more readily. Our experiments demonstrate that **i)** simple greedy vaccine allocation algorithms outperform the natural baseline algorithms substantially (up to 9M more individuals saved than the worst-performing baseline in some settings), **ii)** for both MaxCasesAverted and MaxPeaksReduced, greedy algorithms perform near-optimally for most problem instances we evaluate for New Hampshire (and recover similar approximation guarantees to those of submodular functions for experiments in Iowa and Texas), and **iii)** the fastest of our greedy algorithms are feasible even for large scale instances such as the state of Texas.

## Materials and methods

### Background

**Notation.** We use ${\mathbb{Z}}_{+}$ to denote the set of non-negative integers and for any positive integer *n*, we use [*n*] to denote the set {1,2,…,n}.

**Metapopulation disease-spread models.** A *metapopulation* disease-spread model [11] generalizes the classic homogeneous-mixing compartmental models [27] by allowing geographically-diverse subpopulations. Let $K\in {\mathbb{Z}}_{+}$ denote the number of subpopulations in the metapopulation model. For each subpopulation i∈[K], let *n*_*i*_ denote the size of the subpopulation and let **n** denote the vector $\left(n_{1},n_{2},\ldots ,n_{K}\right)$ of subpopulation sizes. For each pair (i,j)∈[K]×[K], let $w_{ij}\in {\mathbb{Z}}_{+}$ denote the number of individuals moving from subpopulation *i* to subpopulation *j* daily. Thus, each *w*_*ij*_ is a static (i.e., time independent) quantity. Let **W** denote the K×K mobility matrix induced by the *w*_*ij*_ values.

Our goal is to decrease the spread of disease by allocating a total of $D\in {\mathbb{Z}}_{+}$ bundles of vaccines to individuals over all subpopulations; here *D* is the *vaccine budget*. A bundle can be viewed as the smallest “shipment” of vaccines that can be allocated to a subpopulation and we assume that each bundle consists of an integer Δ>0 number of individual vaccines. Let $x=(v_{1},...,v_{K})\in {\mathbb{Z}}_{+}^{K}$ denote a vaccine allocation, where $v_{i}$ is the number of bundles of vaccines allocated to subpopulation *i*. For simplicity, we assume that vaccination is preemptive, i.e., occurs at time 1, with knowledge of initial infected, but before the disease has started to spread. It is straightforward to generalize this to a setting in which vaccine allocation occurs later in the progression of the disease. Let $𝐈=(I_{1}^{0},I_{2}^{0},\ldots ,I_{K}^{0})\in {\mathbb{Z}}_{+}^{K}$, where $0\leq I_{i}^{0}\leq n_{i}$, denote the number of initial infections in subpopulation *i*. Let f(x|ℳ,𝐈) denote some measure of disease-spread according to the metapopulation model ℳ starting with initial infection vector **I**, expressed as a function of the vaccine allocation vector x. For example, f(𝐱|ℳ,𝐈) could denote the total number of infected individuals over some time window. Let g(𝐱|ℳ,𝐈) denote f(0|ℳ,𝐈)−f(𝐱|ℳ,𝐈), representing the reduction in disease-spread due to vaccine allocation $x\in {\mathbb{Z}}_{+}^{K}$, relative to the no-vaccine setting. Note that both *f* and *g* are defined over the integer lattice ${\mathbb{Z}}_{+}^{K}$ and our goal is to maximize the integer lattice function g(x|ℳ,𝐈) subject to the cardinality constraint $\|x{\|}_{1}\leq D$.

**Submodularity of lattice functions** For $K\in {\mathbb{Z}}_{+}$, let $g:{\mathbb{Z}}_{+}^{K}\to \mathbb{R}$ be a function defined on an integer lattice domain. The function *g* is said to be *submodular* if for all $x,y\in {\mathbb{Z}}_{+}$

g(x)+g(y)≥g(x∨y)+g(x∧y) (1)

Here $\left(x\vee y{)}_{i}=\max\{x_{i},y_{i}\right\}$ and $\left(x\wedge y{)}_{i}=\min\{x_{i},y_{i}\right\}$.

Below we provide an alternate “diminishing returns” notion of submodularity that is easier to work with. Here $e_{i}$ denotes the unit vector with 1 in coordinate *i*.

**Definition 1.** *[21] **(DR-Submodularity)** A function $g:{\mathbb{Z}}_{+}^{K}\to \mathbb{R}$ is said to be diminishing returns submodular (DR-submodular, in short) if $g(x+e_{i})-g(x)\geq g(y+e_{i})-g(y)$ for all i∈[K] and $x,y\in {\mathbb{Z}}_{+}^{K}$, where x≤y.*

For set functions, submodularity and DR-submodularity are equivalent. However, it is known [20] that if a lattice function is DR-submodular then it is submodular, but the converse is false. Thus, DR-submodularity is a stronger notion compared to submodularity. However, [24] presents a DR-type characterization of submodular lattice functions that is quite useful for our analysis.

**Lemma 2.** *[24] A function $g:{\mathbb{Z}}_{+}^{K}\to \mathbb{R}$ is submodular if and only if for any $x,y\in {\mathbb{Z}}_{+}^{K}$, x≤y and i∈[K] with $x_{i}=y_{i}$, $g(x+e_{i})-g(x)\geq g(y+e_{i})-g(y)$.*

Note that according to this lemma, for submodular lattice functions, the DR property is only required to hold at identical coordinates of x and y. The computational complexity of maximizing a submodular lattice function $g:{\mathbb{Z}}_{+}^{K}\to \mathbb{R}$ subject to a cardinality constraint, namely $\max_{\|x{\|}_{1}\leq D}g(x)$, is well understood. [20] extend the result for set functions from [28] to lattice functions and show that greedy approaches yield a $\left(1\;-\;\frac{1}{e}\right)$-approximation for this problem for both submodular and DR-submodular lattice functions (an algorithm is said to achieve an α-approximation for a maximization problem if it always produces a solution whose objective value is at least an α fraction (0≤α≤1) of the optimal value). These approximation guarantees are optimal due to the inapproximability result of [29], meaning that no approximation algorithm can achieve a higher constant-factor guarantee.

### The SEIR metapopulation model

The SEIR equations are governed by parameters λ, η, and δ, where λ is the *infectivity*, 1/η is the *latency period*, and 1/δ is the *infectious period*. Let *r*_*i*_ denote a multiplier that scales λ to allow for county differences in contact rates. Let *T* be a positive integer denoting the size of the time window under consideration. For t∈[T]∪{0}, each subpopulation is split into compartments $S_{i}^{t}$, $E_{i}^{t}$, $I_{i}^{t}$, and $R_{i}^{t}$ representing the number of susceptible, exposed, infected, and recovered individuals within subpopulation *i* at time *t*. We assume the initial conditions $E_{i}^{0}=R_{i}^{0}=0$, $I_{i}^{0}$ is an arbitrary non-negative number satisfying $I_{i}^{0}\leq n_{i}$, and $S_{i}^{0}=n_{i}\;-\;I_{i}^{0}$. The evolution of $S_{i}^{t}$, $E_{i}^{t}$, $I_{i}^{t}$, and $R_{i}^{t}$ over time *t* is respectively governed by Eqs 2–5. The term $q_{i}^{t}$ that appears in these equations is called the *force of infection*. When $q_{i}^{t}=\lambda r_{i}\frac{I_{i}^{t}}{n_{i}}$, Eqs 2–5 represent the spread of disease in a single subpopulation *i* with a homogeneous mixing assumption.

$$S_{i}^{t+1}=S_{i}^{t}-q_{i}^{t}S_{i}^{t}$$ (2)

$$E_{i}^{t+1}=E_{i}^{t}+q_{i}^{t}S_{i}^{t}-\eta E_{i}^{t}$$ (3)

$$I_{i}^{t+1}=I_{i}^{t}+\eta E_{i}^{t}-\delta I_{i}^{t}$$ (4)

$$R_{i}^{t+1}=R_{i}^{t}+\delta I_{i}^{t}$$ (5)

We use the following expression for the force of infection term $q_{i}^{t}$ that takes the infection incidence within subpopulation *i* along with flows of individuals into and out of subpopulation *i*. The derivation of $q_{i}^{t}$ is inspired by a similar derivation in [5,12] and is included in Section A in S1 Text.

$$q_{i}^{t}=\lambda \left[r_{i}\left(1-\sum_{j}\frac{w_{ij}}{n_{i}}\right)\frac{{\hat{I}}_{i}^{t}}{{\hat{n}}_{i}}+\sum_{j}\frac{w_{ij}r_{j}}{n_{i}}\frac{{\hat{I}}_{j}^{t}}{{\hat{n}}_{j}}\right]$$ (6)

${\hat{n}}_{i}$ denotes the *effective* population of subpopulation *i* at time *t*, describing the number of individuals present in subpopulation *i* after a daily commute has occurred, and ${\hat{I}}_{i}^{t}$ denotes the effective number of infected individuals in subpopulation *i* after a commute. The first term in the right hand side of Eq 6 is the proportion of individuals leaving subpopulation *i* for their commute, and the second term is the proportion of individuals arriving.

The SEIR metapopulation model ℳ described above is completely specified by the vector (𝐧,𝐫,𝐖,T,λ,η,δ). In our experiments, each subpopulation represents a county within a state (e.g., *K* = 99 for Iowa) and the mobility matrix **W** is obtained from two independent sources, FRED [30] and SafeGraph [31]. By instantiating a specific disease-spread model for each subpopulation and describing its interaction with mobility matrix **W**, we can obtain a completely specified metapopulation model.

Table 1 summarizes the notation introduced in this section.

**Table 1:** Metapopulation model notation.

Variable	Definition
*K*,*T*	Number of subpopulations, size of time window
*r*_*i*_	Population density correlated λ-multiplier for subpopulation *i*
*n*_*i*_	Size of subpopulation *i*
*w*_*ij*_	Mobility from subpopulations *i* to *j*
$q_{i}^{t}$	Force of infection in subpopulation *i* at time *t*
λ,1/η,1/δ	Infectivity, latency period, infectious period

### Problem formulations

We are now ready to state the *Metapopulation Vaccine Allocation* (MVA) family of problems.

**MVA**

Given a metapopulation model ℳ, initial infected vector $𝐈=(I_{1}^{0},I_{2}^{0},\ldots ,I_{K}^{0})\in {\mathbb{Z}}_{+}^{K}$, where $0\leq I_{i}^{0}\leq n_{i}$, and a vaccine budget $D\in {\mathbb{Z}}_{+}$, find a vaccine allocation $x\in {\mathbb{Z}}_{+}^{K}$, satisfying $\|x{\|}_{1}\leq D$ such that g(x|ℳ,𝐈):=f(0|ℳ,𝐈)−f(𝐱|ℳ,𝐈) is maximized.

**SEIR metapopulation vaccine allocation problems.** For illustrative purposes, we instantiate the general metapopulation model ℳ with an SEIR model for disease spread within each subpopulation. Our framework is general and the SEIR model that we use within subpopulations can be replaced by any other homogeneous-mixing disease spread model.

Using the SEIR metapopulation model described above, we obtain specific instances of the MVA problem. But before we can describe these specific instances, we need to describe how vaccination affects disease spread in the SEIR metapopulation model. For simplicity, we assume that vaccine uptake and vaccine effectiveness are both perfect, and thus allocating a vaccine bundle $x=(v_{1},\ldots ,v_{K})$ implies that $\Delta \cdot v_{i}$ individuals in subpopulation *i* are vaccinated and removed from $S_{i}^{0}$. Thus the vaccine allocation $x=(v_{1},\ldots ,v_{K})$ updates the initial susceptible to $S_{i}^{0}=\max(0,n_{i}-I_{i}^{0}-\Delta \cdot v_{i})$ for all i∈[K]. The assumptions of perfect uptake and effectiveness are easily relaxed; lowering the vaccine uptake or effectiveness is equivalent to allocating fewer vaccines.

We now present two illustrative problems that maximize the impact of vaccines according to different disease spread metrics. In the problem MaxCasesAverted, the metric is the total number of infections averted across all subpopulations, and in the problem MaxPeaksReduced, the metric is the decrease in the sum of all infection peaks across all subpopulations (both taken over the entire simulation time). More precisely, given an SEIR metapopulation model ℳ=(𝐧,𝐫,𝐖,T,λ,η,δ), initial infected vector $𝐈=(I_{1}^{0},I_{2}^{0},\ldots ,I_{K}^{0})\in {\mathbb{Z}}_{+}^{K}$, where $0\leq I_{i}^{0}\leq n_{i}$, and a vaccine allocation $x=(v_{1},\ldots ,v_{K})\in {\mathbb{Z}}_{+}^{K}$, we define the metric


$$\text{tot}B\text{urden}(𝐱|ℳ,𝐈):=\sum_{k\in [K]}(R_{k}^{T}+I_{k}^{T}),$$

which is simply the total number of individuals who became infected in the time window [0,*T*]. Another natural disease spread metric for the SEIR metapopulation model is


$$\text{max}B\text{urden}(𝐱|ℳ,𝐈):=\sum_{k\in [K]}\max_{0\leq t\leq T}I_{k}^{t},$$

which is the total number of individuals infected during “peak” infection time over all the subpopulations. This metric is motivated by the fact that even small peaks are challenging in low-resource counties (typically in low-population counties), because healthcare infrastructure is often limited in such counties. So even a small spike in the number of infected individuals can quickly overwhelm local resources. Thus we seek to reduce the likelihood that local healthcare systems will be overwhelmed with the maxBurden metric.

Given metapopulation model ℳ, initial infection vector I, and budget *D*, we define the following discrete optimization problems:

**MaxCasesAverted**

Find a vaccine allocation $x\in {\mathbb{Z}}_{+}^{K}$, satisfying $\|x{\|}_{1}\leq D$ such that the following is maximized.

totBurden(0|ℳ,𝐈)−totBurden(𝐱|ℳ,𝐈)

**MaxPeaksReduced**

Find a vaccine allocation $x\in {\mathbb{Z}}_{+}^{K}$, satisfying $\|x{\|}_{1}\leq D$ such that the following is maximized.

maxBurden(0|ℳ,𝐈)−maxBurden(𝐱|ℳ,𝐈)

### Hardness of MaxCasesAverted and MaxPeaksReduced

As with many resource allocation problems, both MaxCasesAverted and MaxPeaksReduced are not only NP-hard, but even hard to efficiently approximate (additional background on NP-hardness may be found in [32]). The purpose of this section is to formally establish that the MVA problem is too hard to solve exactly in general, making it necessary to use approximation algorithms. We show this by a reduction from the *Maximum k-Subset Intersection* (Max *k*-SI) problem [33]. The input to Max *k*-SI consists of a collection $𝒞=\{P_{1},P_{2},\ldots ,P_{m}\}$ of sets, where each set *P*_*j*_ is a subset of a universe $𝒰=\{p_{1},p_{2},\ldots ,p_{n}\}$, and a positive integer *k*. The problem seeks to find *k* subsets $P_{j_{1}},P_{j_{2}},\ldots ,P_{j_{k}}$ from 𝒞, whose intersection has maximum size. The following theorem from [33] shows that Max *k*-SI is highly unlikely to have an efficient approximation algorithm, even with an inverse polynomial approximation factor.

**Theorem 3.** *[33] Let ϵ>0 be an arbitrarily small constant. Assume that SATISFIABILITY does not have a probabilistic algorithm that decides whether a given instance of size n is satisfiable in time $2^{n^{\epsilon }}$. Then there is no polynomial time algorithm for Max k-SI that achieves an approximation ratio of $1/N^{\epsilon^{\prime }}$, where N is the size of the given instance of Max k-SI and $\epsilon^{\prime }$ only depends only on ϵ.*

We now show a reduction from Max *k*-SI to both MaxCasesAverted and MaxPeaksReduced, thereby establishing the inapproximability of both of these problems.

**Theorem 4.** *Let ϵ>0 be an arbitrarily small constant. Assume that SATISFIABILITY does not have a probabilistic algorithm that decides whether a given instance of size n is satisfiable in time $2^{n^{\epsilon }}$. Then there is no polynomial time algorithm for MaxCasesAverted or for MaxPeaksReduced that achieves an approximation ratio of $1/N^{\epsilon^{\prime }}$, where N is the size of the given instance of MaxCasesAverted or MaxPeaksReduced and $\epsilon^{\prime }$ only depends only on ϵ.*

**Proof:** To prove the portion of this theorem pertaining to MaxCasesAverted, we show the following lemma.

**Lemma 5.** *Suppose there is a polynomial-time algorithm 𝒜 that yields an α-approximation for MaxCasesAverted. Then there is a polynomial-time α/2-approximation algorithm ${𝒜}^{\prime }$ for Max k-SI.*

**Proof of Lemma 5.** Given an instance (𝒞,𝒰,k) of Max *k*-SI, we construct the graph *G* with m+n+1 nodes. For each subset $P_{j}\in 𝒞$ and each $p_{i}\in 𝒰$, there is a node in *G*, for a total of m+n nodes. There is an extra node *I* that is connected to every *P*_*j*_-node. There are edges between the *P*_*j*_-nodes and the *p*_*i*_-nodes connecting an *P*_*j*_-node to an *p*_*i*_-node iff $p_{i}\notin P_{j}$.

To each node *v* in *G*, we assign a population $n_{v}$ as follows: *n*_*I*_ = *m*, $n_{P_{j}}=2n$ for all j∈[n], and $n_{p_{i}}=M$ for all i∈[n], where *M* is a large integer whose value will be specified later.

**Fig 1:**
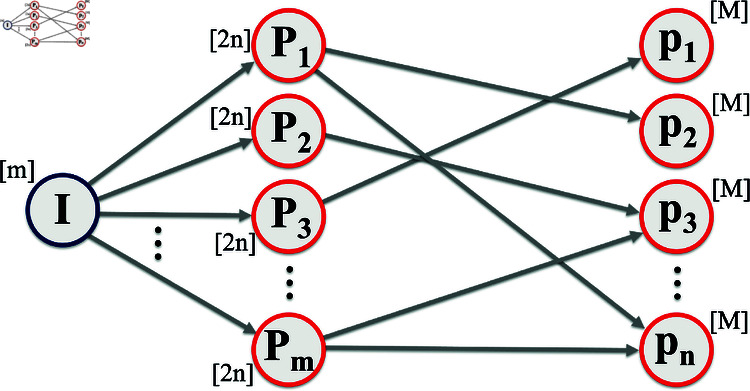
The instance of MaxCasesAverted and MaxPeaksReduced is a graph *G* constructed from the given instance $\left(𝒞=(P_{1},P_{2},\ldots ,P_{m}),𝒰=(p_{1},p_{2},\ldots ,p_{n}),k\right)$ of Max k-SI. Each node represents a subpopulation, with the size of the subpopulation shown in square brackets next to it. The directed edges permit 1 unit flow. The unit flows from nodes *P*_*j*_ to *p*_*i*_ encode non-membership. For example, the flow from *P*_1_ to *p*_2_ implies that $p_{2}\notin P_{1}$.

We then interpret each undirected edge in *G* as a pair of directed edges pointing in opposite directions and assign a flow to each directed edge. We assign flow 1 to each edge from *I* to *P*_*j*_ and to each edge from *P*_*j*_ to *p*_*i*_. To all other edges, i.e., the edges pointing “backwards”, we assign flow 0. This construction is illustrated in Fig 1. This specifies the vectors n and w of the instance of MaxCasesAverted.

We set the contact rate $r_{v}$ and infectivity λ such that the force of infection $q_{v}^{t}$ is always at least 1. This corresponds to “perfect infectivity”, meaning that if a subpopulation contains some infected and some susceptible individuals at a time step, then *all* the susceptible individuals in the subpopulation will transition to the exposed state at the next time step. We then set η=δ=1 so that the latency period and recovery period are both 1. With this setting of the parameters, the infection will completely die out in 5 time steps, i.e., every individual will either be susceptible or recovered. So we set the size of the time window *T* = 5.

Finally, we set the vaccination budget D=(m−k)·2n and initialize the entire population of *m* individuals at node *I* to be infected and all other individuals to be susceptible. This completes the specification of the problem instance ℐ of MaxCasesAverted.

We now make 2 simple observations that follow from the construction of ℐ and depend on the notion of being “unprotected” with respect to a vaccine allocation. Let **x** be an arbitrary, feasible allocation for ℐ. A subpopulation *P*_*j*_ is called *unprotected for* ***x*** if ${𝐱}_{P_{j}}<2n$; otherwise, *P*_*j*_ is called *protected for* ***x***. A subpopulation *p*_*i*_ is called *unprotected for* **x** if ${𝐱}_{p_{i}}<M$ and for some subpopulation *P*_*j*_ that is unprotected for **x**, the edge $\left\{P_{j},p_{i}\right\}$ is in *G*; otherwise, *p*_*i*_ is called *protected for* ***x***.

**Observation 1:** In every unprotected subpopulation *P*_*j*_, j∈[m], $2n\;-{𝐱}_{P_{j}}$ individuals will become exposed in time step 1 and infected in time step 2.

**Observation 2:** In every unprotected subpopulation *p*_*i*_, i∈[n], $M-{𝐱}_{p_{i}}$ individuals will become exposed in time step 3 and infected in time step 4.

These 2 observations immediately lead to the following 3 claims.

**Claim i)** Consider a vaccine allocation $x\in {\mathbb{Z}}_{+}^{m+n+1}$ that is feasible for ℐ and satisfies $x_{p_{i}}>0$. Let $x^{\prime }\in {\mathbb{Z}}_{+}^{m+n+1}$ be an allocation obtained from **x** by reallocating all vaccines from the subpopulation *p*_*i*_ to subpopulations *P*_*j*_, j∈[m]. Then ${𝐱}^{\prime }$ is feasible for ℐ and


$$\text{tot}B\text{urden}({𝐱}^{\prime }|ℳ,𝐈)\leq \text{tot}B\text{urden}(𝐱|ℳ,𝐈).$$

**Claim ii)** Consider a vaccine allocation $x\in {\mathbb{Z}}_{+}^{m+n+1}$ that is feasible for ℐ and satisfies $0<x_{P_{j}},x_{P_{j^{\prime }}}<2n$ for two subpopulations $P_{j},P_{j^{\prime }}$, $j\notin =j^{\prime }$. Let $x^{\prime }\in {\mathbb{Z}}_{+}^{m+n+1}$ be an allocation obtained from **x** by reallocating as many vaccines as possible from the subpopulation $P_{j^{\prime }}$ to the subpopulation *P*_*j*_, until $x_{P_{j}}=2n$ or $x_{P_{j^{\prime }}}=0$ (or both). Then ${𝐱}^{\prime }$ is feasible for ℐ and


$$\text{tot}B\text{urden}({𝐱}^{\prime }|ℳ,𝐈)\leq \text{tot}B\text{urden}(𝐱|ℳ,𝐈).$$

**Claim iii)** Consider a vaccine allocation $x\in {\mathbb{Z}}_{+}^{m+n+1}$ that is feasible for ℐ and satisfies $||x|{|}_{1}=D=(m\;-\;k)\;\cdot \;2n$. Then using the reallocations from Claims (i) and (ii), it is possible to transform **x** into ${𝐱}^{\prime }\in {\mathbb{Z}}_{+}^{m+n+1}$ in polynomial time such that ${𝐱}^{\prime }$ is feasible for ℐ, ${𝐱}_{P_{j}}^{\prime }=2n$ for exactly (*m*–*k*) subpopulations *P*_*j*_, ${𝐱}^{\prime }$ is 0 for all other subpopulations, and


$$\text{tot}B\text{urden}({𝐱}^{\prime }|ℳ,𝐈)\leq \text{tot}B\text{urden}(𝐱|ℳ,𝐈).$$

Claim (iii) allows us to assume that any α-approximation algorithm 𝒜 for MaxCasesAverted returns an allocation ${𝐱}^{\prime }$ for the problem instance ℐ, that picks exactly (*m*–*k*) subpopulations *P*_*j*_ and vaccinates these subpopulations entirely, while allocating no vaccines to any of the remaining subpopulations. Similarly, Claim (iii) implies that there is an optimal allocation ${𝐱}^{*}$ for ℐ that picks exactly (*m*–*k*) subpopulations *P*_*j*_ and vaccinates these subpopulations entirely, while allocating no vaccines to any of the remaining subpopulations.

Let $𝒮({𝐱}^{\prime })$ be the set of subpopulations *P*_*j*_ unprotected for ${𝐱}^{\prime }$. Similarly, define $𝒮({𝐱}^{*})$. Note that $|𝒮({𝐱}^{\prime })|=|𝒮({𝐱}^{*})|=k$. Let $ℰ({𝐱}^{\prime })$ be the set of subpopulations *p*_*i*_ that are protected for ${𝐱}^{\prime }$. Similarly, define $ℰ({𝐱}^{*})$. By the construction of edges from subpopulations *P*_*j*_ to subpopulations *p*_*i*_ in ℐ, it follows that $ℰ({𝐱}^{\prime })=\cap_{P_{j}\in 𝒮({𝐱}^{\prime })}P_{j}$. Similarly, $ℰ({𝐱}^{*})=\cap_{P_{j}\in 𝒮({𝐱}^{*})}P_{j}$

The objective function value of MaxCasesAverted for the optimal allocation ${𝐱}^{*}$, which is $\text{tot}B\text{urden}(0|ℳ,𝐈)-\text{tot}B\text{urden}({𝐱}^{*}|ℳ,𝐈)$, can be simplified to


$$\left(2n\cdot m+n\cdot M)-\text{tot}B\text{urden}({𝐱}^{*}|ℳ,𝐈\right)$$

$$=(2n\cdot m+n\cdot M)-(k\cdot 2n+M(n-|ℰ({𝐱}^{*})|))$$

$$=2n(m-k)+M\cdot |ℰ({𝐱}^{*})|$$

$$=2n(m-k)+M\cdot |\cap_{P_{j}\in 𝒮({𝐱}^{*})}P_{j}|$$ (7)

Similarly, the objective function value of MaxCasesAverted for the α-approximate allocation ${𝐱}^{\prime }$ is $2n(m\;-\;k)\;+\;M\;\cdot \;|\;\cap_{P_{j}\in 𝒮({𝐱}^{\prime })}\;P_{j}|$. Since ${𝐱}^{*}$ maximizes the objective function value of MaxCasesAverted, Eq 7 implies that $\left|\;\cap_{P_{j}\in 𝒮({𝐱}^{*})}\;P_{j}\right|$ has largest possible cardinality. Since $|𝒮({𝐱}^{*})|=k$, this implies that $𝒮({𝐱}^{*})$ is an optimal solution to the Max *k*-SI problem. Using $OPT_{M\text{ax}\;k\text{-}\mathrm{SI}}$ to denote the optimal objective function value of Max *k*-SI, we can rewrite the expression (7) as $2n(m\;-\;k)\;+\;M\;\cdot \;OPT_{M\text{ax}\;k\text{-}\mathrm{SI}}$. Since ${𝐱}^{\prime }$ is an α-approximate solution to MaxCasesAverted,


$$2n(m-k)+M\cdot |\cap_{P_{j}\in 𝒮({𝐱}^{\prime })}P_{j}|\geq \alpha \left(2n(m-k)+M\cdot OPT_{M\text{ax}\;k\text{-}\mathrm{SI}}\right).$$

Rearranging terms we get


$$|\cap_{P_{j}\in 𝒮({𝐱}^{\prime })}P_{j}|\geq \alpha \cdot OPT_{M\text{ax}\;k\text{-}\mathrm{SI}}-\frac{\left(1-\alpha )\cdot 2n(m-k\right)}{M}$$

$$|\cap_{P_{j}\in 𝒮({𝐱}^{\prime })}P_{j}|\geq \alpha \cdot OPT_{M\text{ax}\;k\text{-}\mathrm{SI}}-\frac{2nm}{M}$$ (8)

Picking *M* large enough so that $\frac{2nm}{M}\leq \frac{\alpha }{2}$ and using $OPT_{M\text{ax}\;k\text{-}\mathrm{SI}}\geq 1$, we obtain


$$|\cap_{P_{j}\in 𝒮({𝐱}^{\prime })}P_{j}|\geq \frac{\alpha }{2}\cdot OPT_{M\text{ax}\;k\text{-}\mathrm{SI}}.$$

This implies that the allocation ${𝐱}^{\prime }$ can be used to obtain an α/2-approximation to Max *k*-SI.

We now prove a similar lemma for the MaxPeaksReduced problem.

**Lemma 6.** *Suppose there is a polynomial-time algorithm 𝒜 that yields an α-approximation for MaxPeaksReduced. Then there is a polynomial-time α-approximation algorithm ${𝒜}^{\prime }$ for Max k-SI.*

**Proof of Lemma 6.** This uses the same argument as the lemma above. Claims (i) and (ii) hold for maxBurden(𝐱|ℳ,𝐈) as well and from these two claims, Claim (iii) follows. Furthermore,


$$\text{max}B\text{urden}(0|ℳ,𝐈)-\text{max}B\text{urden}({𝐱}^{*}|ℳ,𝐈)$$

simplifies exactly to expression (7), from which inequality (8) follows. From this, the lemma immediately follows, as shown above.

### Algorithmic approach and analysis

We present the following four greedy algorithms for MVA:

1. ℓ**-EnumGreedy**: Enumerates all feasible solutions with at most ℓ subpopulations allocated a positive number of vaccine bundles. Then each of these solutions is iteratively extended greedily, one subpopulation at a time, with a variable number of additional vaccine shipments. Because of the potentially large number of initial feasible solutions, this is only suitable for small and medium-scale problems.
2. **SingletonGreedy**: Finds one solution by extending the empty solution greedily, one subpopulation at a time, until *D* shipments are allocated. Compares this solution to the *K* solutions by allocating the entire budget *D* to each of the *K* subpopulations. Returns the best of these *K* + 1 solutions.
3. **FastGreedy**: Runs a “relaxed” version of greedy that stops its search as soon as it finds a “good enough” additional allocation of vaccine shipments. The threshold for a “good enough” allocation is adaptive, i.e., may change from iteration to iteration. This algorithm trades off solution quality for speed and is suitable for large-scale problems.
4. **UnitGreedy**: Starting from the empty allocation, greedily allocates just one vaccine shipment at a time. Searching over a space of just one additional vaccine shipment per subpopulation, speeds up each iteration. This algorithm is suitable for large-scale problems.

In the following, we first describe these algorithms in further detail and then we establish approximation guarantees for ℓ-EnumGreedy, SingletonGreedy, and UnitGreedy based on how close the objective function is to being submodular. These algorithms and their accompanying analyses also apply to the general budget-constrained maximization problem on an integer lattice: $\max_{\|x{\|}_{1}\leq D}g(x)$, where $g:{\mathbb{Z}}_{+}^{K}\to \mathbb{R}$ is an arbitrary, monotone function defined on an integer lattice.

We start by defining the LatticeGreedySubroutine, whose search space is the entire lattice ${\mathbb{Z}}_{+}^{K}$ in each iteration. This subroutine forms the basis for two greedy algorithms ℓ-EnumGreedy and SingletonGreedy [20] (detailed below). Algorithms based on LatticeGreedySubroutine are prohibitively expensive for large problem instances, so we also consider FastGreedy [25], which is a relaxation of LatticeGreedySubroutine, based on a threshold greedy algorithm [34]. In addition, we evaluate and further analyze an approach which considers the lattice as a multiset and runs the greedy algorithm for set functions over it, which we call UnitGreedy (Algorithm 3). In this section, we describe each algorithm we evaluate and their associated approximation guarantees, some of which we derive.

### Greedy algorithm descriptions

#### LatticeGreedySubroutine description

We first describe LatticeGreedySubroutine, which is the core component of ℓ-EnumGreedy and SingletonGreedy. The foundation of LatticeGreedySubroutine is to repeatedly apply a “locally optimal” approach, where the subpopulation and number of vaccine shipments is applied that would improve the objective function the most.

As shown in the Algorithm 1 pseudocode, LatticeGreedySubroutine selects a $\left({\text{k}}^{*},{\text{s}}^{*}\right)$ pair that maximizes the marginal gain of g(·) in each iteration, where $k^{*}\in [K]$ is a subpopulation and $s^{*}\in {\mathbb{Z}}_{+}$ is the number of bundles to allocate to subpopulation ${\text{k}}^{*}$. To compute the highest marginal gain among all possible $(k,s)\in [K]\;\times \;{\mathbb{Z}}_{+}$ pairs in each iteration of the algorithm, we assume that the algorithm has access to a “value oracle” that returns the value of the objective function g(·) at any point in its domain. It is possible that the selected pair $\left({\text{k}}^{*},{\text{s}}^{*}\right)$ is not feasible because adding it to the solution causes the budget constraint to be violated. Such an iteration is said to have *failed*, and we remove the $\left({\text{k}}^{*},{\text{s}}^{*}\right)$ pair from the search space *Q*, which is a list that LatticeGreedySubroutine maintains of all feasible allocations. Otherwise, the iteration is *successful* and the $\left({\text{k}}^{*},{\text{s}}^{*}\right)$ pair is used to update the allocation. It is useful for our analysis to state the algorithm in this manner, allowing for failed iterations. However, to obtain an efficient implementation we can, in Line 4, prune the search space *Q* so as to guarantee that the condition in Line 5 is always satisfied. Such an implementation runs in $O(K\;\cdot \;D^{2}\;\cdot \;T_{g})$ time in the worst case, where *T*_*g*_ is the worst case running time of the value oracle. However, the at most K·D pairs in *Q* can all be evaluated in parallel, and assuming full parallelism with no overhead, the running time of LatticeGreedySubroutine can also be reduced to $O(D\;\cdot \;T_{g}v\log(K\;\cdot \;D))$ in the PRAM model (even with exclusive read and exclusive write). We note that LatticeGreedySubroutine and the algorithms based on it come from [20].

#### ℓ-EnumGreedy description

We further allow LatticeGreedySubroutine to start with an arbitrary initial allocation ${\hat{x}}_{0}$, and not just 0 (see Line 1). This is so that we can use LatticeGreedySubroutine as the completion step for an algorithm that enumerates solutions of bounded size. Specifically, let ℓ≥1 be a fixed integer and let 𝒮 be the set of all feasible solutions of size ℓ or less. Thus each element in 𝒮 is a subset of at most ℓ subpopulations, each allocated some number of vaccine bundles so that the overall allocation is of size at most *D*. Note that $\left|𝒮|=O(K^{\ell }\cdot D^{\ell }\right)$. We then iterate over all elements of 𝒮 and call LatticeGreedySubroutine with ${\hat{x}}_{0}$ set to each element in 𝒮. We call this entire algorithm ℓ-EnumGreedy. Later in this section, we analyze 3-EnumGreedy.

#### SingletonGreedy description

While 3-EnumGreedy runs in polynomial time (specifically, $O(K^{4}\;\cdot \;D^{5}\;\cdot \;T_{g})$ time), it is expensive and not practical for large instances. A cheaper algorithm based on LatticeGreedySubroutine computes one solution by starting LatticeGreedySubroutine with 0 as the initial allocation and then computes *K* additional “singleton” solutions by allocating the entire budget to each of the *K* subpopulations. The final solution returned is the best of these *K* + 1 solutions. We call this the SingletonGreedy algorithm. Note that the running time of SingletonGreedy is dominated by LatticeGreedySubroutine.

**Algorithm 1** LatticeGreedySubroutine ($ℳ,𝐈,{\hat{x}}_{0}$)

1: $\hat{x}\leftarrow {\hat{x}}_{0}$

2: $Q:=\{(k,s):k\in [K],1\leq s\leq \lceil \frac{n_{k}}{\Delta }\rceil -{\hat{x}}_{k}\}$

3: **while** $\|\hat{x}{\|}_{1}<D$ and Q≠∅ **do**

4:   $k^{*},s^{*}\leftarrow \underset{(k,s)\in Q}{argmax}\;\frac{g(\hat{x}+s\cdot e_{k}|ℳ,𝐈)-g(\hat{x}|ℳ,𝐈)}{s}$

5:   **if** $\|\hat{x}+s^{*}\cdot e_{k^{*}}{\|}_{1}\leq D$ **then**

6:    $\hat{x}\leftarrow \hat{x}+s^{*}\cdot e_{k^{*}}$

7:    $Q\leftarrow Q\;\backslash \;\{(k,s):s+{\hat{x}}_{k}>\lceil \frac{n_{k}}{\Delta }\rceil \}$

8:   **else**

9:    Remove (k*, s*) from *Q*

10:   **end if**

11: **end while**

12: **return** $\hat{x}$

#### FastGreedy description

FastGreedy [25] is a more flexible version of LatticeGreedySubroutine that maintains a threshold value to determine how strict the algorithm is in choosing allocations. FastGreedy starts with a high threshold ($\tau_{f}$) value that determines the minimum benefit required from a (*k*,*s*) pair to select it, and $\tau_{f}$ is relaxed as FastGreedy progresses. In each iteration, any (*k*,*s*) pair that provides benefit above the current threshold gets selected, allowing multiple selections per iteration (unlike LatticeGreedySubroutine, which picks only one). At the end of each iteration, the threshold is lowered according to the rate parameters $\kappa_{f}$ and $\delta_{f}$. More specifically, $\kappa_{f}$ controls how much $\tau_{f}$ decreases in each iteration, where a higher value of $\kappa_{f}$ will result in higher standards for selection in each iteration. $\delta_{f}$ controls how quickly $\beta_{f}$ approaches $\beta_{f}^{*}$, which is an upper-bound on the DR-submodularity ratio (see Eq 7 and [25]). The parameter $\varepsilon_{f}$ determines the minimum progress FastGreedy must make in order to terminate early (i.e., before the budget is met).

FastGreedy differs from LatticeGreedySubroutine in two ways that make it more efficient: i) FastGreedy allows allocation to multiple subpopulations in a single iteration instead of only one per iteration, and ii) FastGreedy determines the best number of bundles through a binary search subroutine (included in Appendix B) instead of searching through every (*k*,*s*) pair (which LatticeGreedySubroutine does).

#### UnitGreedy description.

On the problem instances we consider, in practice, 3-EnumGreedy, and SingletonGreedy elect to allocate one bundle at a time for a majority of iterations. With this in mind, we consider another more efficient algorithm, UnitGreedy. As shown in the Algorithm 3 pseudocode, UnitGreedy allocates one vaccine bundle to a subpopulation k∈[K], each time selecting a subpopulation that yields the highest marginal gain in the objective function - this is equivalent to converting the lattice into a multiset and running a set greedy algorithm on it (such as the one in [26]). The algorithm continues until the vaccine budget *D* is met.

The running time of this algorithm is $O(K\;\cdot \;D\;\cdot \;T_{g})$. Note that the marginal gains for the various bundles can be computed (Line 3 in Algorithm 3) in parallel in a straightforward manner, and if we ignore overhead for parallelization, the running time reduces to $O(D\cdot T_{g})$.

**Algorithm 2** FastGreedy($ℳ,𝐈,\kappa_{f},\delta_{f},\varepsilon_{f}\in (0,1)$)

1: $x\leftarrow 0,M\leftarrow \underset{k\in [K]}{\max}\;g(e_{k}),m\leftarrow M,m^{\prime }\leftarrow M/\kappa_{f},\beta_{f}\leftarrow 1$

2: **while** $m\geq M\varepsilon_{f}/D$ **do**

3:   $m\leftarrow \underset{k\in [K]}{\max}\;g(x+e_{k})-g(x)$

4:   **if** $m>\kappa_{f}m^{\prime }$ **then**

5:    $\beta_{f}\leftarrow \beta_{f}\delta_{f}$

6:   **end if**

7:   $m^{\prime }\leftarrow m$

8:   $\tau_{f}\leftarrow \beta_{f}\kappa_{f}m$

9:   **for** k∈[K] **do**

10:    $\ell \leftarrow \text{BinarySearchPivot}(ℳ,g,x,k,D,\tau_{f})$

11:    $x\leftarrow x+\ell e_{k}$

12:    **if** $\|x{\|}_{1}=D$ **then return** x

13:    **end if**

14:   **end for**

15: **end whilereturn** x

**Algorithm 3** UnitGreedy(ℳ,𝐈)

1: $\hat{x}\leftarrow 0$

2: **while** $\|\hat{x}{\|}_{1}<D$ **do**

3:   $k^{*}\leftarrow \underset{k\in [K]}{argmax}\;g(\hat{x}+e_{k}|ℳ,𝐈)-g(\hat{x}|ℳ,𝐈)$

4:   $\hat{x}\leftarrow \hat{x}+e_{k^{*}}$

5: **end while**

6: **return** $\hat{x}$

### Approximation guarantees

The performance of our algorithms depends on how close to submodularity their objective functions are. In this section, we i) formally define the notion of “distance to submodularity” and ii) connect these definitions to the quality of output of our algorithms.

#### Lattice function submodularity ratios

To analyze the greedy algorithms described above, we utilize the notion of *submodularity ratio* defined in [24]. The submodularity ratio of a function *g* is a quantity between 0 and 1 that is a measure of *g*’s “distance” to submodularity. Since there are two distinct notions of submodularity for lattice functions, as defined in the Background section, there are two associated notions of submodularity ratios, which we now present. To simplify notation, we drop ℳ and **I** and simply use g(x) for our objective function.

**Definition 7. DR-Submodularity Ratio.** *[24] The DR-submodularity ratio of a function $g:{\mathbb{Z}}_{+}^{K}\to \mathbb{R}$ is defined as*

$$\beta (g)=\min_{y\leq x,k\in [K]}\frac{g(y+e_{k})-g(y)}{g(x+e_{k})-g(x)}$$ (9)

In this definition (and in the next definition below) we designate $\frac{0}{0}$ to be 1 and $\frac{n}{0}$ to be ∞ for any positive integer *n*. From this definition it is clear that β(g)≤1 because x=y is included in the space that is being minimized over. Furthermore, this definition along with the definition of DR-submodularity (Definition 1) implies that β(g)=1 iff *g* is DR-submodular. Thus, the “distance” 1−β(g) indicates how far the function *g* is from being DR-submodular. Below we present a similar definition that captures the notion of “distance” of a function *g* from being submodular.

**Definition 8. Submodularity Ratio.** *[24] The submodularity ratio of a function $g:{\mathbb{Z}}_{+}^{K}\to \mathbb{R}$ is defined as*

$$\alpha (g)=\min_{y\leq x,k\in [K]:x_{k}=y_{k}}\frac{g(y+e_{k})-g(y)}{g(x+e_{k})-g(x)}$$ (10)

Like β(g), the submodularity ratio α(g) also satisfies α(g)≤1 and 1−α(g) indicates how far the function *g* is from being submodular. Since submodularity is a weaker notion than DR-submodularity, an arbitrary lattice function will be “closer” to submodularity than DR-submodularity. Correspondingly, α(g)≥β(g).

We now present approximation guarantees for 3-EnumGreedy (Theorem 9a), SingletonGreedy (Theorem 9b), and UnitGreedy (Theorem 10). The approximation guarantee associated with FastGreedy can be found in [25].

#### Guarantees for 3-EnumGreedy and SingletonGreedy

Theorem 9 provides a guarantee for 3-EnumGreedy and SingletonGreedy. Previously, [20] established approximation guarantees for these algorithms over submodular objective functions, whereas we establish them for more general objective functions.

**Theorem 9.** *Let $g:{\mathbb{Z}}_{+}^{K}\to \mathbb{R}$ be an arbitrary monotone function. Let OPT denote the optimal solution to the problem $\max_{\|x{\|}_{1}\leq D}g(x)$.*

*(a) If $\hat{x}$ is the solution returned by* 3-EnumGreedy *then*


$$g(\hat{x})\geq (1-e^{-\alpha (g)})\cdot OPT$$

*(b) If $\hat{x}$ is the solution returned by* SingletonGreedy *then*


$$g(\hat{x})\geq \frac{\alpha (g)}{2}\cdot (1-e^{-\alpha (g)})\cdot OPT$$

The proof is included in Section B.1 in S1 Text.

#### Guarantee for UnitGreedy

Here, we provide a version of the approximation guarantee found in [26], which is dependent on the submodularity ratio for set functions [23] and generalized curvature [26]. Their guarantee is applicable to UnitGreedy when we consider the lattice over which we allocate to be a multiset.

**Theorem 10.** *Let $g:{\mathbb{Z}}_{+}^{K}\to \mathbb{R}$ be an arbitrary monotone function. Let OPT denote the optimal solution to the problem $\max_{\|x{\|}_{1}\leq D}g(x)$. If $\hat{x}$ is the solution returned by* UnitGreedy *then*


$$g(\hat{x})\geq (1-e^{-\beta (g)})\cdot OPT$$

The above results show that the approximation guarantees shown in the literature [20,28] for greedy algorithms when *g* is a submodular function (on sets or lattices) are more general and apply to arbitrary monotone integer lattice functions.

Note that the theorems above also provide a trade-off between approximation-factor and running time. UnitGreedy is the fastest algorithm, but this provides an $\left(1\;-\;e^{-\beta (g)}\right)$-approximation, which is no better than the $\left(1\;-\;e^{-\alpha (g)}\right)$-approximation provided by the more expensive algorithm 3-EnumGreedy.

We remark that ℓ-EnumGreedy, SingletonGreedy, FastGreedy, and UnitGreedy are well known algorithms for maximizing a submodular function over sets or lattices subject to a cardinality constraint (e.g., [19,20,25,26]). Our main contribution here is to show that 3-EnumGreedy and SingletonGreedy provide approximation guarantees even when the objective function is not submodular and these guarantees degrade gracefully as the objective function becomes less submodular, as measured by the submodularity ratio. We also derive a lattice function based approximation guarantee for UnitGreedy, extending from the set function guarantee provided in [26].

**Table 2:** Summary of greedy algorithms presented in this section. Details of approximation factors for FastGreedy and UnitGreedy may be found in [26] and [25], respectively.

Algorithm	Query Complexity	Approximation Factor	Prior Work
3-EnumGreedy	$O(K^{4}\cdot D^{5})$	$1\;-\;e^{-\alpha (g)}$ [this paper]	Analysis for latticeeak submodular functions [19,20]
SingletonGreedy	$O(K^{2}\cdot D^{2})$	$\frac{\alpha (g)}{2}(1\;-\;e^{-\alpha (g)})$ [this paper]	
FastGreedy	$O((\log_{\delta_{f}}(\gamma_{d})\;\cdot \;\log_{\kappa_{f}}(\gamma_{d})$ $+\log_{\kappa_{f}}\varepsilon^{2}/D)\cdot K\log D)$	$1\;-\;e^{-\kappa_{f}\beta^{*}\gamma_{s}}\;-\;\varepsilon$ [prior work]	Analysis for latticeeak non-submodular functions [25]
UnitGreedy	O(K·D)	$\frac{1}{\alpha_{g}}(1\;-\;e^{-\alpha_{g}\gamma_{dk}})$ [prior work] $1\;-\;e^{-\beta (g)}$ [this paper]	Analysis for multiset non-submodular functions [26]

Finally, we note that the POMS algorithm in [24] achieves a $\max((1\;-\;e^{-\beta (g)}),\alpha (g)/$$2\cdot (1-e^{-\alpha (g)}))$ -approximation. Our results show that simple, well-known, and faster greedy algorithms achieve these same approximation factors.

## Results

Next, we present a variety of experiments that collectively show that **i)** greedy methods outperform various baseline vaccine allocation algorithms for both MaxCasesAverted and MaxPeaksReduced objectives, **ii)** greedy methods are very close to optimal for all instances for which this comparison was feasible, and **iii)** the greedy methods are considerably faster than POMS [24] (when requiring all algorithms to run until their approximation factors can be guaranteed).

We run our experiments at 3 different scales: (i) small-scale experiments: New Hampshire (10 counties, population 1.4 million), (ii) medium-scale experiments: Iowa (99 counties, population 3.2 million), and (iii) large-scale experiments: Texas (254 counties, population 30.03 million).

Our code and processed data are made available. Experiments were run on AMD EPYC 7763 CPUs with 2 TB RAM.

### Baselines

Our baselines include natural vaccine allocation strategies such as Population, Out-Mobility, In-Mobility, and Random, which assign vaccines to each county proportional to the population, the total mobility originating in the county, the total mobility terminating in the county, and uniformly at random respectively. We also compare our approaches against POMS [24], which works by expanding a random pareto-optimal frontier.

### Data

Our experimental test-beds consist of simulated outbreaks over inter-county mobility graphs for New Hampshire, Iowa and Texas constructed from two separate sources:

**FRED (Framework for Reconstructing Epidemic Dynamics)** [30] (open source) includes a census-based synthetic population with high-resolution social, familial, demographic, and behavioral details. We infer a daily-commute mobility network from home and work locations.

- **Movement**: Daily inter-county commute statistics.
- **Coverage**: Uniform coverage ensuring that home and work locations for every county (i.e., subpopulation) are modeled to an equal degree.
- **Strengths**: Captures essential work-based mobility and is consistent across counties.
- **Limitations**: Lacks recreational, shopping, and irregular mobility patterns.

**SafeGraph** [31] (open source for academics) provides aggregated and anonymized mobility data from mobile device GPS signals, which provides inferred ‘home’ locations and visits to places of interest (POIs).

- **Movement**: All types of travel including work, leisure, shopping, social visits, and other activities.
- **Coverage**: Broad coverage for urban areas with comprehensive mobile and internet infrastructure.
- **Strengths**: Captures a comprehensive view of mobility including irregular patterns.
- **Limitations**: Potentially unreliable coverage for rural subpopulations due to lower mobile phone infrastructure.

**Mobility graphs.** We derive state-level directed mobility graphs from both data sources, where nodes correspond to counties and directed weighted edges correspond to movement from the source county to the target county, with edge weights representing the number of commutes between county pairs. The mobility graphs constructed using FRED and SafeGraph are similar for New Hampshire and Iowa (the SafeGraph mobility graphs have a slightly higher density). The New Hampshire graphs are nearly complete due to the state’s small size (it can be crossed by vehicle in about 45 minutes). In contrast, the mobility graphs for Texas reveal significant differences between FRED and SafeGraph data sources. The density of the FRED mobility graph is an order of magnitude lower than that of SafeGraph. This difference occurs because SafeGraph captures more irregular travel patterns, including instances where individuals travel long distances across Texas. Such cross-state travel is relatively rare compared to New Hampshire, where short distances make travel between any two points feasible, but it takes nearly 10 hours to cross Texas by vehicle. A more comprehensive description of mobility graph construction and a table of their properties appears in Section C.2 and Table A in S1 Text.

### Parameters

We select values of λ (infectivity) at approximately 0.347 and 0.535 to result in 20% and 70% of each population becoming infected without vaccination, respectively. We conducted experiments with a wider range of λ values (in general, we observed that problem instances with lower values of λ are more easily solved by more vaccine allocation methods) and chose two values that represent significantly different levels of infectivity. We performed experiments for New Hampshire, Iowa, and Texas, with a vaccine budget of 10% through 60% of each state’s total population in 10% increments. Each vaccine budget refers to the total percent of the population for which vaccines are available, and we assume that the entire vaccine budget will be used up for each budget amount. The parameters *k*, *n*_*i*_, and *w*_*ij*_ are instantiated according to the data when we constructed the mobility graphs. The parameters *r*_*i*_ scale the infectivity parameter λ for each county, and is set in proportion to the population density of each county. We set the initially infected vector **I** to be 1 for each county. The choice in **I** does not make a difference in our setting due to the deterministic nature of our model and the small diameter of our mobility graphs (at most 4). η and δ are set according to [35]. For FastGreedy in New Hampshire and Iowa, we set $\kappa_{f}=\delta_{f}=0.96$, and in Texas, we set $\kappa_{f}=\delta_{f}=0.93$. For all FastGreedy experiments, we set $\varepsilon_{f}=0$. We run each simulation for at least 200 timesteps and terminate the simulation when the disease dies out.

### Performance of greedy methods compared to baselines

**Fig 2:**
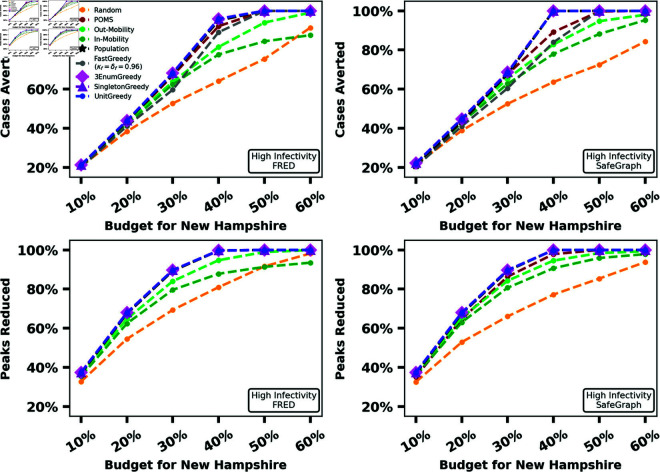
Percentage totBurden and percentage maxBurden reduced by all approaches for λ = 0.5345 in New Hampshire for FRED (first column) and SafeGraph (second column).

**Fig 3:**
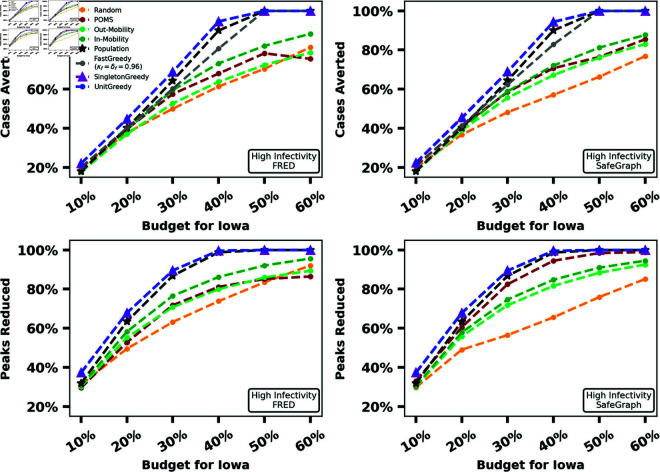
Percentage totBurden and percentage maxBurden reduced by UnitGreedy, SingletonGreedy, FastGreedy and baselines for λ = 0.535 in Iowa for FRED (first column) and SafeGraph (second column).

**Fig 4:**
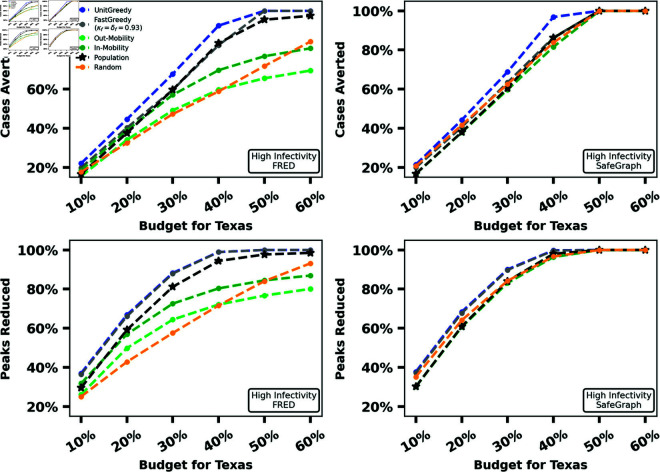
Percentage totBurden and percentage maxBurden reduced by UnitGreedy, FastGreedy and baselines for λ = 0.525 in Texas for FRED (first column) and SafeGraph (second column).

In our first experiment, we compare the performance of greedy vaccine allocation algorithms to baseline algorithms using both the FRED and SafeGraph mobility graphs, for both the MaxCasesAverted and MaxPeaksReduced problems. For our small-scale experiment (New Hampshire), we run all four greedy algorithms. For our medium-scale experiment (Iowa), we drop our slowest greedy algorithm 3-EnumGreedy because the initial enumeration step required by 3-EnumGreedy too prohibitively expensive the number of subpopulations grows. For our large-scale experiment (Texas), we drop our slowest two greedy algorithms (3-EnumGreedy and SingletonGreedy) because exploring every possible number of shipments to each subpopulation in a reasonable amount of time is infeasible for this scale. For this comparison, we always run POMS for the same amount of time as UnitGreedy. We seek to demonstrate how close the performance of POMS gets to that of UnitGreedy in a simple wall clock time based comparison. We repeat these experiments for six different budgets (expressed as a percentage of the population of the state) for two different values of λ. The results for a high infectivity value of λ are summarized in Figs 2, 3, and 4. The same experiments for lower infectivity parameter values can be found in Section C.3 in S1 Text.

Fig 2 shows that, for our small-scale experiments, the baselines never outperform the greedy methods. Population and POMS perform on-par with the greedy methods in some instances, particularly in MaxPeaksReduced. We see the performance of baselines relative to the greedy methods decline as the scale of our experiments become larger.

As observed in Fig 3, even for our medium-scale experiments, the greedy algorithms outperform each baseline in several settings, while no baseline outperforms the greedy methods. Fig 4 demonstrates that, for our large-scale experiment, UnitGreedy and FastGreedy outperform the baselines by a wider margin than our small and medium-scale experiments over the FRED dataset. This margin is more narrow (with UnitGreedy and FastGreedy still in the lead) over the SafeGraph mobility graph. For SafeGraph, UnitGreedy and FastGreedy perform on-par with the same methods over the FRED data - the difference is primarily in the increased performance of the baselines over SafeGraph. We note that for our large-scale experiment (Texas), the mobility graph constructed using SafeGraph data is far more dense than the one constructed using FRED data, and as a result, disease flows much more freely over the subpopulations. This highlights how mobility graph structure can impact the effectiveness of simple vaccine allocation methods. Similar results hold for a lower value of λ, which can be found in Figs B, C, and D in S1 Text.

UnitGreedy performs at least on-par with the other greedy methods, all of which employ larger search spaces. In all experiments, after the greedy methods, the Population heuristic performs well, followed by POMS, other baselines, and finally Random. The relatively poor performance of POMS could be attributed to the fact that it requires a long running time to achieve its theoretical guarantee (see Performance-Time Trade-off). The surprisingly good performance of the Population heuristic suggests that it might be a good on-the-field strategy in the absence of mobility data for small problem instances. UnitGreedy substantially outperforms Population and FastGreedy for our large-scale experiment on Texas over FRED data, with totBurden reduced by up to 8% of the population, which translates to almost 2 million additional cases avoided.

### Near-optimality of greedy algorithms

In this section, we demonstrate that in practice, the greedy algorithms we evaluate return allocations whose objective function value is close to optimal for both MaxCasesAverted and MaxPeaksReduced. First, we directly compute optimal solutions for our small-scale experiment (New Hampshire) using mobility derived from FRED data, which would be prohibitively expensive for our medium-scale and large-scale settings. We consider 4 problem instances for each of MaxCasesAverted and MaxPeaksReduced, obtained by setting λ to 0.347 and 0.5345 and the budget *D* to 2 values (10% and 40% of the population). For these problem instances we compute an optimal solution by exhaustive search and compare the results to that of 3-EnumGreedy, SingletonGreedy, UnitGreedy, and FastGreedy.

For each problem and problem instance, let OPT denote the objective function value of an optimal solution. Table 3 shows the performance relative to OPT of problem instances for 10% and 40% budgets, high and low infectivity, and both objective functions for each greedy method.

**Table 3:** Approximation factors for each problem instance.

**NH (FRED) 10% Budget**	Cases Averted		Peaks Reduced	
	λ=0.3475	λ=0.5355	λ=0.3475	λ=0.5355
3-EnumGreedy	99.84%	98.53%	99.71%	96.33%
SingletonGreedy	79.02%	99.04%	99.71%	99.33%
FastGreedy	79.02%	95.29%	92.29%	95.21%
UnitGreedy	79.02%	99.04%	99.71%	96.33%
**NH (FRED) 40% Budget**	Cases Averted		Peaks Reduced	
	λ=0.3475	λ=0.5355	λ=0.3475	λ=0.5355
3-EnumGreedy	100%	99.86%	100%	99.97%
SingletonGreedy	100%	99.86%	100%	99.97%
FastGreedy	100%	99.26%	100%	99.97%
UnitGreedy	100%	99.86%	100%	99.97%

Problem instances for the state of Iowa are much larger and it is not feasible to compute OPT to make a direct comparison. To circumvent this problem, we first note that it is possible to obtain improved versions of Theorems 9(a), 9(b), and 10 by defining “per instance” versions of the DR-submodularity ratio and submodularity ratio. To be specific, let ${\hat{x}}_{i}$ denote the allocation after iteration *i* of UnitGreedy, let $x^{*}$ be an optimal solution, and let $y^{*}=0\vee (x^{*}-{\hat{x}}_{i})$. Define

$$\beta (g,{\hat{x}}_{i}):=\frac{\sum_{j=1}^{K}g(y_{j}^{*}e_{j}+{\hat{x}}_{i})-g({\hat{x}}_{i})}{g(y^{*}+{\hat{x}}_{i})-g({\hat{x}}_{i})}$$ (11)

The numerator is the total marginal gain of independently increasing each individual subpopulation’s allocation to the optimal allocation. The denominator is the marginal gain of increasing ${\hat{x}}_{i}$ to the optimal solution all at once. If *g* were submodular, it would follow that $\beta (g,{\hat{x}}_{i})\geq 1$, but this guarantee does not hold for an arbitrary g(·). It is possible to show that the bound stated in Theorem 10 holds for $\beta (g,\hat{x})$, i.e., $g(\hat{x})\geq (1-e^{-\beta (g,\hat{x})})\cdot OPT$ (more on this may be found in Section C.4 in S1 Text).

Since we cannot calculate the optimal solution $x^{*}$ directly (and $\beta (g,{\hat{x}}_{i})$ depends on $x^{*}$) we cannot calculate $\beta (g,{\hat{x}}_{i})$ directly either. Instead, we use a sampling method (described in Section C.4 in S1 Text) to find an estimate of $\beta (g,{\hat{x}}_{i})$, which we denote as $\hat{\beta }(g,{\hat{x}}_{i})$. We calculate $\hat{\beta }(g,{\hat{x}}_{i})$ 5000 times for each experiment to estimate $\beta (g,{\hat{x}}_{i})$. Our key finding is that $\hat{\beta }(g,\hat{x})$ is very close to (or even larger than) 1 for most of our experimental instances, implying that *g* might be close to being submodular in practice. This suggests that the allocation $g(\hat{x})\geq (1-1/e)\cdot OPT\approx 0.63\cdot OPT$.

Estimates for $\beta (g,\hat{x})$ can be found in Table 4. These values indicate worst-case approximation factors for performance on-par (and some exceeding) that of submodular functions for our problem formulations and experimental settings.

**Table 4:** Estimates of $\beta (g,\hat{x})$ for each problem instance.

**NH (FRED) 60% Budget**	Cases Averted		Peaks Reduced	
	λ=0.3475	λ=0.5355	λ=0.3475	λ=0.5355
3-EnumGreedy	1.03	1.01	1.74	1.02
SingletonGreedy	1.41	1.01	1.53	1.02
FastGreedy	1.04	1.01	1.05	1.01
UnitGreedy	1.51	1.02	2.66	1.12
**IA (FRED) 60% Budget**	Cases Averted		Peaks Reduced	
	λ=0.3475	λ=0.5355	λ=0.3475	λ=0.5355
SingletonGreedy	1.02	1.01	1.06	1.01
FastGreedy	1.04	1.01	1.05	1.01
UnitGreedy	1.02	1.02	1.07	1.04
**TX (FRED) 60% Budget**	Cases Averted		Peaks Reduced	
	λ=0.3475	λ=0.5355	λ=0.3475	λ=0.5355
FastGreedy	1.12	1.03	1.04	1.03
UnitGreedy	1.02	1.02	1.06	1.03

### Performance and running-time trade-offs

Here, we compare the performance and running time trade-offs for 3-EnumGreedy, SingletonGreedy, FastGreedy, UnitGreedy and POMS. Let $c_{\max}=\max\{n_{i}|i\in [K]\}$. The approximation guarantee for POMS requires $T=2ec_{\max}D^{2}K$ queries [24]; this makes POMS significantly more expensive to run compared to the greedy methods. The term “query” refers to an evaluation of the objective function g(·); here, that evaluation entails running a disease simulation conditioned on a vaccine allocation. Compared to POMS, UnitGreedy requires relatively fewer T=K·D queries. In addition, UnitGreedy is much faster in practice (by Wall Clock Time) than POMS since UnitGreedy is embarrassingly parallel, whereas POMS is much more inherently sequential. These comparisons are presented in Table 5, where we list required iterations and practical run time (extrapolated from 12 hours for POMS). FastGreedy introduces an approximation guarantee parameterized by a value which upper bounds the DR-submodularity ratio. Their input parameters can be adjusted to determine the quality required of potential allocation in each iteration, effectively trading performance for speed. When the input parameters to FastGreedy are set so that the performance is maximized, the resulting approximation guarantee is similar to that of UnitGreedy, 3-EnumGreedy, and SingletonGreedy.

**Table 5:** FastGreedy, UnitGreedy, SingletonGreedy, 3-EnumGreedy, and POMS comparison with respect to practical running time (estimated for POMS) to achieve approximation guarantee for New Hampshire with 20% and 60% budgets.

**NH (FRED) 20% Budget**	Queries Required		Wall Clock Time	
	λ=0.3475	λ=0.5355	λ=0.3475	λ=0.5355
FastGreedy	1567	70	3.9 Minutes	7.4 Seconds
UnitGreedy	$6.67\cdot 10^{3}$	$6.67\cdot 10^{3}$	16.7 Minutes	7.6 Minutes
SingletonGreedy	$6.43\cdot 10^{5}$	$7.57\cdot 10^{5}$	1 Hour	37.1 Minutes
3-EnumGreedy	$6.53\cdot 10^{5}$	$7.58\cdot 10^{5}$	1 Hour	37.6 Minutes
POMS	$3.63\cdot 10^{15}$	$3.63\cdot 10^{15}$	$∼1.5\cdot 10^{8}$ Years	$∼1.2\cdot 10^{8}$ Years
**NH (FRED) 60% Budget**	Queries Required		Wall Clock Time	
	λ=0.3475	λ=0.5355	λ=0.3475	λ=0.5355
FastGreedy	4761	2025	7.7 Minutes	3.3 Minutes
UnitGreedy	$2\cdot 10^{4}$	$2\cdot 10^{4}$	33.2 Minutes	30 Minutes
SingletonGreedy	$3.54\cdot 10^{6}$	$2.7\cdot 10^{6}$	3.9 Hours	3.4 Hours
3-EnumGreedy	$3.55\cdot 10^{6}$	$2.66\cdot 10^{6}$	5.1 Hours	1.5 Hours
POMS	$3.27\cdot 10^{16}$	$3.27\cdot 10^{16}$	$∼1.1\cdot 10^{9}$ Years	$∼1.1\cdot 10^{9}$ Years

## Discussion

Through a combination of theoretical and experimental results, we have shown that even though metapopulation model vaccine allocation problems are inapproximable in the worst case, simple greedy algorithms can be both effective and scalable for these problems. We provide a possible theoretical explanation for the effectiveness of these greedy algorithms by establishing worst case approximation guarantees in terms of the submodularity ratios of the objective functions of these problems. Specifically, we extend worst case approximation guarantees from the literature for lattice greedy algorithms [20,25,26] to the non-submodular objective function setting. Our analysis builds upon prior work on submodular set and lattice function maximization [5,10,19,20,28,34].

For specific instantiations of the metapopulation model vaccine allocation problems (e.g., MaxCasesAverted, MaxPeaksReduced) we provide some empirical evidence that the submodularity ratio of the objective functions is high enough (i.e., close enough to 1) to imply that greedy algorithms yield near-optimal solutions to these problems. The greedy algorithms we evaluate are effective across small (New Hampshire), medium (Iowa), and large (Texas) problem scales over two mobility graphs constructed from FRED [30] and SafeGraph [36] data sources. In all problem instances of MVA we evaluate, the greedy methods outperform the baselines, sometimes by quite a significant margin. This difference in performance is typically greatest for a high λ (infectivity) value, vaccinating 30% to 50% of the total state’s population for each problem scale. We also demonstrate that the greedy algorithms achieve an approximation factor of over 0.79 for a 10% budget, and an approximation factor of over 0.99 with a 40% budget for both MaxCasesAverted and MaxPeaksReduced problem instances over New Hampshire.

We observe the performance of the greedy methods are on-par with each other for the Texas FRED and SafeGraph mobility graphs, but the performance of the baselines over the FRED mobility graph are much lower. Because of this, we conjecture that the MVA problem over sparse mobility graphs is harder to solve and we cannot depend on the baselines. Across all experiments, we observe that the MVA problem instances with a lower infectivity value λ - infecting approximately 20% of the population - are generally easier to achieve good performance on for all methods.

Moreover, we have parallelized our algorithms to enhance scalability. As a result, the fastest of our algorithms takes 2-3 hours to run for the state of Texas. The ability to parallelize the computation allows us to manage the computational demands of large states, ensuring that our methods remain feasible even in large-scale datasets. The query complexities for each greedy algorithm (shown in Table 2) further contributes to the feasibility and speed of the fastest two greedy algorithms we present, UnitGreedy and FastGreedy. In addition, it is quite natural to speed up greedy methods by looking not just for a locally optimal update in each iteration, but for an *approximately* optimal update, which is a main principle behind the threshold approach of FastGreedy. These features of the greedy methods present a computational advantage with respect to scalability over algorithms such as POMS, introduced in [24].

Despite these contributions, several limitations remain. Our current disease model is relatively simple and deterministic and it assumes homogeneous mixing within subpopulations. Our model can be extended in a variety of ways in order to better capture the complexities of real-world disease spread. It would relatively easy to extend the SEIR model we use to allow for additional compartments (e.g., an infected but asymptomatic compartment). Another simple extension for future work could be to extend the model to one that captures certain demographics (e.g., age, gender, etc.) of the population, such as the one presented in [7]. Incorporating demographics of the population into the disease model would be the first step in designing a vaccine allocation method that prioritizes certain groups. A more granular approach would be to incorporate agent-based simulations within subpopulations, to better reflect heterogeneous contact patterns. The choice of a disease model in our setting is largely driven by the needs of the vaccine allocation methods. On the other hand, the granularity and sophistication of the disease model has a direct impact on the computational cost of our vaccine allocation methods. This trade-off, between sophistication of the disease models and the computational cost of the vaccine allocation methods should be carefully considered when choosing a disease model. Exploring faster, more scalable algorithms, such as sketch-based methods [25,37], could alleviate this trade-off to some extent and is a promising direction for future research. An additional limitation is that the inferred mobility data we use is based on limited sources and does not fully reflect real-world movement patterns, particularly in rural areas. Expanding to include more comprehensive mobility data, such as transportation networks, would improve accuracy. Our work focuses on preemptive vaccine allocation, i.e., vaccine allocation at the beginning of an outbreak. Expanding our work to consider vaccine allocation over time as the disease spreads and more vaccines become available is also a promising direction for future research. For this paper, we ran experiments on individual states in isolation without taking physical border effects into account, where in real-world settings, the influence of areas (especially urban) across a state border could have significant impact on vaccine allocation decisions. Finally, deriving confidence bounds for the estimated submodularity ratios would enhance the robustness of our theoretical guarantees.

## Supporting information

**Fig A in S1 Text. Iowa and New Hampshire mobility graphs derived from FRED data.** We overlay mobility graphs over maps of Iowa and New Hampshire, where the size of each node is proportional to the population size of the subpopulation in which it is centered. Likewise, the width of each edge $e\in E_{ij}$ is proportional to its weight *w*_*ij*_ (number of individuals commuting from subpopulation *i* to subpopulation *j*).

**Fig B in S1 Text. Percentage MaxCasesAverted and percentage MaxPeaksReduced for all approaches in New Hampshire under low infectivity**. Most methods are able to save all individuals across all budgets for this small problem instance, with Random being the lowest performing method.

**Fig C in S1 Text. Percentage MaxCasesAverted and percentage MaxPeaksReduced for UnitGreedy, SingletonGreedy, FastGreedy and baselines in Iowa under low infectivity.** The effectiveness of the greedy methods is largely unchanged from that of the small problem instances (New Hampshire), but the baseline methods begin to decrease in performance.

**Fig D in S1 Text. Percentage MaxCasesAverted and percentage MaxPeaksReduced for UnitGreedy, FastGreedy and baselines in Texas under low infectivity.** For the SafeGraph mobility graph, all methods are able to save most individuals for all budgets, unlike for the FRED mobility graph, where the performance decreases for smaller budgets.

S1 Text Contains Supplementary Information sections A–D, detailing model derivation, approximation guarantee proofs, descriptions of mobility graph construction, parameters, additional experiments, and related work.
(PDF)

**Table A in S1 Text. Comparison of FRED and SafeGraph mobility graph properties.** Contains properties of the mobility graphs constructed from FRED and SafeGraph data in New Hampshire, Iowa, and Texas.

**Table B in S1 Text. System specifications for experiments.** Contains information on the CPU type, memory, and storage where we run experiments.

**Table C in S1 Text. Metapopulation model notation.** Summary of the notation used for the metapopulation disease model.

**Table D in S1 Text. Problem formulations and algorithm notation.** Summary of the notation used for problem formulations and algorithms.

(PDF)
