# Supplementary material for: Analyzing greedy vaccine allocation algorithms for metapopulation disease models
Source: PLoS Comput Biol. 2025 Jul 21;21(7):e1012539. doi: 10.1371/journal.pcbi.1012539 (PMC12289052; doi:10.1371/journal.pcbi.1012539)
Supplement: S1 Text — Contains Supplementary Information sections A–D, detailing model derivation, approximation guarantee proofs, descriptions of mobility graph construction, parameters, additional experiments, and related work. (PDF) [file pcbi.1012539.s001.pdf]

Supporting information

A Derivation of q_i^t

Let $\hat{I}_i^t$ represent the *effective number of infected individuals* (after movement) in each subpopulation. Then, Eq 1 describes the evolution of $\hat{I}_i^t$.

$$\hat{I}_i^t = I_i^t + \sum_j \frac{w_{ji}}{n_j} I_j^t - \sum_j \frac{w_{ij}}{n_i} I_i^t \tag{1}$$

We define the *effective population* using Eq 2.

$$\hat{n}_i = n_i + \sum_j (w_{ji} - w_{ij}) \tag{2}$$

Then the rate of infection for susceptible individuals from subpopulation i who did not move is:

$$\lambda r_i \left(S_i^t - S_i^t \sum_j \frac{w_{ij}}{n_i} \right) \frac{\hat{I}_i^t}{\hat{n}_i} \tag{3}$$

The rate of infection for susceptible individuals in subpopulation i who moved into subpopulation j is:

$$\lambda \left(S_i^t \sum_j \frac{w_{ij} r_j}{n_i} \frac{\hat{I}_j^t}{\hat{n}_j} \right) \tag{4}$$

By combining Eqs 3 and 4 into one term representing the total rate at which susceptible individuals from subpopulation i get infected, we get the following expression for q_i^t:

$$q_i^t = \lambda \left[r_i \left(1 - \sum_j \frac{w_{ij}}{n_i} \right) \frac{\hat{I}_i^t}{\hat{n}_i} + \sum_j \frac{w_{ij} r_j}{n_i} \frac{\hat{I}_j^t}{\hat{n}_j} \right] \tag{5}$$

B Algorithmic analysis

B.1 Proofs of theorems

BinarySearchPivot is a subroutine used by FastGreedy to determine the best number of vaccines to send to subpopulation k, and is used by FastGreedy for each subpopulation $k \in [K]$. The purpose is to find the highest number ℓ of vaccines to send to k such that the average marginal gain of g exceeds the current threshold value τ_f.

Theorem 9. *Let $g : \mathbb{Z}_+^K \to \mathbb{R}$ be an arbitrary monotone function. Let OPT denote the optimal solution to the problem $\max_{\|\mathbf{x}\|_1 \leq D} g(\mathbf{x})$.*
*(a) If $\hat{\mathbf{x}}$ is the solution returned by 3-*EnumGreedy *then*

$$g(\hat{\mathbf{x}}) \geq (1 - e^{-\alpha(g)}) \cdot OPT$$

(b) If $\hat{\mathbf{x}}$ is the solution returned by SingletonGreedy *then*

$$g(\hat{\mathbf{x}}) \geq \frac{\alpha(g)}{2} \cdot (1 - e^{-\alpha(g)}) \cdot OPT$$

Algorithm 1 BinarySearchPivot$(\mathcal{M}, g, \mathbf{x}, k, D, \tau_f)$

$\ell_s \leftarrow 1, \ell_t \leftarrow \min\{\mathbf{n}_k - \mathbf{x}_k, D - \|\mathbf{x}\|_1\}$
if $g(\mathbf{x} + \ell_t \mathbf{e_k} \mid \mathcal{M}, \mathbf{I}) - g(\mathbf{x} \mid \mathcal{M}, \mathbf{I}) \geq \ell_t \tau_f$ **then**
    return ℓ_t
end if
if $g(\mathbf{x} + \mathbf{e_k} \mid \mathcal{M}, \mathbf{I}) - g(\mathbf{x} \mid \mathcal{M}, \mathbf{I}) \leq \tau_f$ **then**
    return 0
end if
while $\ell_t \neq \ell_s + 1$ **do**
    $m \leftarrow \lfloor (\ell_t + \ell_s)/2 \rfloor$
    if $g(\mathbf{x} + m\mathbf{e_k} \mid \mathcal{M}, \mathbf{I}) - g(\mathbf{x} \mid \mathcal{M}, \mathbf{I}) \geq m\tau_f$ **then**
        $\ell_s \leftarrow m$
    else
        $\ell_t \leftarrow m$
    end if
end while

return ℓ_s

Proving Theorem 9 Suppose $OPT = \{(k_1, s_1), (k_2, s_2), \ldots, (k_t, s_t)\}$, where the (subpopulation, allocation)-pairs are arranged in non-decreasing order of marginal gain. Consider a run of LatticeGreedySubroutine in which $\hat{\mathbf{x}}$ is initialized to $\{(k_1, s_1), (k_2, s_2), (k_3, s_3)\}$. Note that since 3-EnumGreedy considers all feasible solutions of size 3 or less, this is one of the executions of LatticeGreedySubroutine considered by 3-EnumGreedy. For this execution of LatticeGreedySubroutine, let $L \in \mathbb{Z}_+$ be such that iterations 1 through $L - 1$ of LatticeGreedySubroutine are successful, but iteration L fails. If all iterations in LatticeGreedySubroutine are successful, then L is the index of the last iteration. For $1 \leq i \leq L$, let $\hat{\mathbf{x}}_\mathbf{i}$ be the allocation chosen by Algorithm LatticeGreedySubroutine up to (and including) iteration i. Let $\Delta_i^{GG} = g(\hat{\mathbf{x}}_\mathbf{i}) - g(\hat{\mathbf{x}}_{\mathbf{i-1}})$.

We now prove a key lemma, which essentially says that the improvement made in iteration $i + 1$ by LatticeGreedySubroutine is at least a $\frac{\alpha(g)}{D}$-fraction of the gap between OPT and the progress made by LatticeGreedySubroutine in iterations 1 through i. For a submodular function the improvement fraction would have been $\frac{1}{D}$, but for an arbitrary function g, we show that the improvement fraction degrades to $\frac{\alpha(g)}{D}$, i.e., linearly in $\alpha(g)$. We also note that the new DR-type characterization of submodular lattice functions by [1] plays a key role in this proof.

Lemma A1. *For every i, $0 \leq i < L$,*

$$\Delta_{i+1}^{GG} \geq \frac{\alpha(g)}{D} \left(OPT - \sum_{j=1}^{i} \Delta_j^{GG} \right)$$

Proof:

For $\mathbf{x}, \mathbf{y} \in \mathbb{Z}_+^K$, let $g(\mathbf{y} \mid \mathbf{x}) = g(\mathbf{y} + \mathbf{x}) - g(\mathbf{x})$. Let $\mathbf{x}^*$ be an optimal solution; thus $OPT = g(\mathbf{x}^*)$. For $0 \leq i < L$, let $\mathbf{x_i} = \mathbf{0} \vee (\mathbf{x}^* - \hat{\mathbf{x}}_\mathbf{i})$.

Since g is monotone, $g(\mathbf{x_i} + \hat{\mathbf{x}}_\mathbf{i}) \geq OPT$ since $\mathbf{x_i} + \hat{\mathbf{x}}_\mathbf{i} \geq \mathbf{x}^*$. Therefore,

$$g(\mathbf{x_i} \mid \hat{\mathbf{x}}_\mathbf{i}) \geq OPT - g(\hat{\mathbf{x}}_\mathbf{i}) \tag{6}$$

Let $\mathbf{x_i} = (\mathbf{x_{i}}_1, \mathbf{x_{i}}_2, \ldots, \mathbf{x_{i}}_K)$ and for $1 \le j \le K$, let $\mathbf{p_j} = (\mathbf{x_{i}}_1, \ldots, \mathbf{x_{i}}_j, 0, \ldots, 0)$, with $\mathbf{p_0} = \mathbf{0}$. Then we can write $g(\mathbf{x_i} \mid \hat{\mathbf{x}}_\mathbf{i})$ as the telescoping sum

$$g(\mathbf{x_i} \mid \hat{\mathbf{x}}_\mathbf{i}) = \sum_{j=1}^{K} g(\mathbf{p_j} + \hat{\mathbf{x}}_\mathbf{i}) - g(\mathbf{p_{j-1}} + \hat{\mathbf{x}}_\mathbf{i}) \tag{7}$$

We now upper bound each term in this sum via the following claim.
Claim: For $1 \le j \le K$,

$$g(\mathbf{p_j} + \hat{\mathbf{x}}_\mathbf{i}) - g(\mathbf{p_{j-1}} + \hat{\mathbf{x}}_\mathbf{i}) \le \frac{1}{\alpha(g)} \left(g(\mathbf{x_{i}}_j \mathbf{e_j} + \hat{\mathbf{x}}_\mathbf{i}) - g(\hat{\mathbf{x}}_\mathbf{i}) \right)$$

Proof of claim: Writing $\mathbf{p_j} = \mathbf{x_{i}}_j \mathbf{e_j} + \mathbf{p_{j-1}}$ and using $\mathbf{y}$ as a short hand for $\mathbf{p_{j-1}} + \hat{\mathbf{x}}_\mathbf{i}$, we can express the left hand side of the inequality in the claim as the telescoping sum

$$\sum_{\ell=1}^{\mathbf{x_{i}}_j} g(\ell \cdot \mathbf{e_j} + \mathbf{y}) - g((\ell-1) \cdot \mathbf{e_j} + \mathbf{y})$$

Similarly, we can express the right hand side of the inequality in the claim as a telescoping sum

$$\frac{1}{\alpha(g)} \sum_{\ell=1}^{\mathbf{x_{i}}_j} g(\ell \cdot \mathbf{e_j} + \hat{\mathbf{x}}_\mathbf{i}) - g((\ell-1) \cdot \mathbf{e_j} + \hat{\mathbf{x}}_\mathbf{i}).$$

We now note that

$$\begin{aligned} & g(\ell \cdot \mathbf{e_j} + \mathbf{y}) - g((\ell-1) \cdot \mathbf{e_j} + \mathbf{y}) \\ & \le \frac{1}{\alpha(g)} \left(g(\ell \cdot \mathbf{e_j} + \hat{\mathbf{x}}_\mathbf{i}) - g((\ell-1) \cdot \mathbf{e_j} + \hat{\mathbf{x}}_\mathbf{i}) \right) \end{aligned}$$

This follows from the definition of $\alpha(g)$ and the fact that coordinate j in $(\ell-1) \cdot \mathbf{e_j} + \hat{\mathbf{x}}_\mathbf{i}$ and $(\ell-1) \cdot \mathbf{e_j} + \mathbf{y}$ are identical. The claim follows. □
Combining (9) and (10) with the Claim, we get

$$\sum_{j=1}^{K} g(\mathbf{x_{i}}_j \mathbf{e_j} + \hat{\mathbf{x}}_\mathbf{i}) - g(\hat{\mathbf{x}}_\mathbf{i}) \ge \alpha(g)(OPT - g(\hat{\mathbf{x}}_\mathbf{i})) \tag{8}$$

Since each iteration $i+1$, $0 \le i < L$, of LatticeGreedySubroutine is successful, the objective function is improved by a quantity Δ_{i+1}^{GG}, satisfying

$$\Delta_{i+1}^{GG} \ge \max_{1 \le j \le K} \frac{g(\mathbf{x_{i}}_j \mathbf{e_j} + \hat{\mathbf{x}}_\mathbf{i}) - g(\hat{\mathbf{x}}_\mathbf{i})}{\mathbf{x_{i}}_j}$$

Since the maximum on the right hand side of the above inequality is at least as large as the average gain in the objective function value per unit budget, we get

$$\Delta_{i+1}^{GG} \ge \frac{\sum_{j=1}^{K} g(\mathbf{x_{i}}_j \mathbf{e_j} + \hat{\mathbf{x}}_\mathbf{i}) - g(\hat{\mathbf{x}}_\mathbf{i})}{\sum_{j=1}^{K} \mathbf{x_{i}}_j}$$

Since $\sum_{j=1}^{K} \mathbf{x_{i}}_j \le D$, we can combine the above inequality with (8) to get $\Delta_{i+1}^{GG} \ge \frac{\alpha(g)}{D}(OPT - g(\hat{\mathbf{x}}_\mathbf{i}))$. □

Using Lemma A1, we now prove Theorem 9 using standard algebra (as in [2]).

Proving Theorem 9(a) Let $OPT = OPT_1 + OPT_2$, where OPT_1 is the optimal solution value for the first ℓ allocations and OPT_2 is the marginal influence of the remaining allocations. We know $\sum_{i=1}^{\ell} \Delta_i^{GG} = OPT_1$ by assumption according to the enumeration step in 3-EnumGreedy. We start with a lemma and corollary. Let $D = D_1 + D_2$, where D_1 is the budget allocated to the first ℓ pairs and D_2 is the remaining budget.

Lemma A2.

$$OPT_2 - \sum_{j=1}^{i} \Delta_j^{GG} \leq \prod_{j=1}^{i} \left(1 - \frac{\alpha(g)s_j}{D_2}\right) \cdot OPT_2$$

for $0 \leq i \leq L$

Proof: By induction. The base case with $i = 0$ is trivially true. Assume the lemma holds for $i - 1$ where $i \geq 1$. Then

$$\begin{aligned} &OPT_2 - \sum_{j=1}^{i} \Delta_j^{GG} \\ &\leq OPT_2 - \sum_{j=1}^{i-1} \Delta_j^{GG} - \frac{\alpha(g)s_i}{D_2} \left(OPT_2 - \sum_{j=1}^{i-1} \Delta_j^{GG}\right) \\ &\qquad \text{[by Lemma 8]} \\ &= \left(1 - \frac{\alpha(g)s_i}{D_2}\right) \left(OPT_2 - \sum_{j=1}^{i-1} \Delta_j^{GG}\right) \\ &\leq \left(1 - \frac{\alpha(g)s_i}{D_2}\right) \prod_{j=1}^{i-1} \left(1 - \frac{\alpha(g)s_j}{D_2}\right) \cdot OPT_2 \\ &\qquad \text{[by inductive hypothesis]} \\ &= \prod_{j=1}^{i} \left(1 - \frac{\alpha(g)s_j}{D_2}\right) \cdot OPT_2 \end{aligned}$$

□

Corollary A3.

$$\sum_{i=1}^{L} \Delta_i^{GG} \geq (1 - e^{-\alpha(g)}) \cdot OPT_2$$

Proof: Let $\phi = \ln(1 - x)$. Note that ϕ is concave and monotonically decreasing in the range $(0, 1]$. Then for $y_1, ..., y_L \in (0, 1]$, we know $\sum_{i=1}^{L} \frac{\phi(y_i)}{L} \leq \phi\left(\sum_{i=1}^{L} \frac{y_i}{L}\right)$ by

Jensen's Inequality. Let $y_i \leftarrow \frac{\alpha(g)s_i}{D_2}$. Then

$$\begin{aligned}
\frac{1}{L}\sum_{i=1}^{L}\ln\left(1-\frac{\alpha(g)s_i}{D_2}\right) &\leq \ln\left(1-\frac{\alpha(g)\sum_{i=1}^{L}s_i}{LD_2}\right)\\
\Rightarrow \sum_{i=1}^{L}\ln\left(1-\frac{\alpha(g)s_i}{D_2}\right) &\leq \ln\left(1-\frac{\alpha(g)\sum_{i=1}^{L}s_i}{LD_2}\right)^L\\
\Rightarrow \prod_{i=1}^{L}\left(1-\frac{\alpha(g)s_i}{D_2}\right) &\leq \left(1-\frac{\alpha(g)\sum_{i=1}^{L}s_i}{LD_2}\right)^L\\
&\leq \left(1-\frac{\alpha(g)}{L}\right)^L
\end{aligned}$$

Since $\sum_{i=1}^{L} s_i/D_2 \geq 1$
By Lemma A2, we have

$$\begin{aligned}
OPT_2-\sum_{i=1}^{L}\Delta_i^{GG} &\leq \prod_{i=1}^{L}\left(1-\frac{\alpha(g)s_i}{D_2}\right)\cdot OPT_2\\
&\leq \left(1-\frac{\alpha(g)}{L}\right)^L\cdot OPT_2\\
&\leq e^{-\alpha(g)}\cdot OPT_2
\end{aligned}$$

Then $\sum_{i=1}^{L}\Delta_i^{GG} \geq (1-e^{-\alpha(g)})\cdot OPT_2$ □

Now we can prove theorem 9(a).

Proof: Let $ALG = ALG_1 + ALG_2$, where ALG_1 is the solution up to budget D_1 and ALG_2 is the objective value for all allocations following ℓ with respect to ALG_1. We know $ALG_1 = OPT_1$ by assumption.
If 3-EnumGreedy succeeds in iteration L, then $ALG_2 = \sum_{i=1}^{L}\Delta_i^{GG}$. Otherwise, if 3-EnumGreedy fails in iteration L, $ALG_2 \geq \sum_{i=1}^{L-1}\Delta_i^{GG}$. Note that in this case, $\Delta_L^{GG} \leq \alpha(g)\frac{OPT_1}{\ell}$.
Then $ALG_2 \geq \sum_{i=1}^{L}\Delta_i^{GG} - \alpha(g)\frac{OPT_1}{\ell}$.
So

$$\begin{aligned}
g(\hat{\mathbf{x}}) &= ALG = ALG_1 + ALG_2\\
&\geq OPT_1 + \sum_{i=1}^{L}\Delta_i^{GG} - \alpha(g)\frac{OPT_1}{\ell}\\
&\geq \left(1-\frac{\alpha(g)}{\ell}\right)OPT_1 + \left(1-\frac{1}{e^{\alpha(g)}}\right)OPT_2\\
&\quad \text{[by Corollary]}\\
&\geq (1-e^{-\alpha(g)})OPT\\
&\quad \text{[since } \ell \geq 3 \text{ and } \alpha(g) \leq 1\text{]}
\end{aligned}$$

□

Proving Theorem 9 (b)

If allocating the entire budget to a single subpopulation yields a marginal gain of at least $\frac{\alpha(g)}{2}\cdot(1-e^{-\alpha(g)})\cdot OPT$, the guarantee is fulfilled. Otherwise, suppose

SingletonGreedy fails on iteration L, then $ALG \geq \sum_{i=1}^{L-1} \Delta_i^{GG}$. Note that $\Delta_L^{GG} \leq \frac{\alpha(g)}{2}(1 - e^{-\alpha(g)})OPT$. Then

$$
\begin{aligned}
ALG &\geq \sum_{i=1}^{L} \Delta_i^{GG} - \Delta_L^{GG} \\
&\geq \sum_{i=1}^{L} \Delta_i^{GG} - \frac{\alpha(g)}{2}\left(1 - \frac{1}{e^{\alpha(g)}}\right) OPT \\
&\geq \left(1 - \frac{1}{e^{\alpha(g)}}\right) OPT - \frac{\alpha(g)}{2}\left(1 - \frac{1}{e^{\alpha(g)}}\right) OPT \\
&\geq \frac{\alpha(g)}{2}\left(1 - \frac{1}{e^{\alpha(g)}}\right) OPT \quad \text{[since } \alpha(g) \leq 1]
\end{aligned}
$$

□

Theorem 10. *Let $g : \mathbb{Z}_+^K \to \mathbb{R}$ be an arbitrary monotone function. Let OPT denote the optimal solution to the problem $\max_{\|\mathbf{x}\|_1 \leq D} g(\mathbf{x})$. If $\hat{\mathbf{x}}$ is the solution returned by* UnitGreedy *then*

$$g(\hat{\mathbf{x}}) \geq (1 - e^{-\beta(g)}) \cdot OPT$$

Proving Theorem 10. We prove Theorem 10 using a similar argument to the proof of Theorem 9. First, we prove a key lemma, which says that the improvement made in iteration $i+1$ by UnitGreedy is at least a $\frac{\beta(g)}{D}$-fraction of the gap between OPT and the progress made by UnitGreedy in iterations 1 through i.

Lemma A4. *For every i, $0 \leq i < L$,*

$$\Delta_{i+1}^{UG} \geq \frac{\beta(g)}{D}\left(OPT - \sum_{j=1}^{i} \Delta_j^{UG}\right)$$

Proof:

For $\mathbf{x}, \mathbf{y} \in \mathbb{Z}_+^K$, let $g(\mathbf{y} \mid \mathbf{x}) = g(\mathbf{y} + \mathbf{x}) - g(\mathbf{x})$. Let $\mathbf{x}^*$ be an optimal solution; thus $OPT = g(\mathbf{x}^*)$. For $0 \leq i < L$, let $\mathbf{x_i} = \mathbf{0} \vee (\mathbf{x}^* - \hat{\mathbf{x}}_\mathbf{i})$.

Since g is monotone, $g(\mathbf{x_i} + \hat{\mathbf{x}}_\mathbf{i}) \geq OPT$ since $\mathbf{x_i} + \hat{\mathbf{x}}_\mathbf{i} \geq \mathbf{x}^*$. Therefore,

$$g(\mathbf{x_i} \mid \hat{\mathbf{x}}_\mathbf{i}) \geq OPT - g(\hat{\mathbf{x}}_\mathbf{i}) \tag{9}$$

Let $\mathbf{x_i} = (\mathbf{x_i}_1, \mathbf{x_i}_2, \ldots, \mathbf{x_i}_K)$ and for $1 \leq j \leq K$, let $\mathbf{p_j} = (\mathbf{x_i}_1, \ldots, \mathbf{x_i}_j, 0, \ldots, 0)$, with $\mathbf{p_0} = \mathbf{0}$. Then we can write $g(\mathbf{x_i} \mid \hat{\mathbf{x}}_\mathbf{i})$ as the telescoping sum

$$g(\mathbf{x_i} \mid \hat{\mathbf{x}}_\mathbf{i}) = \sum_{j=1}^{K} g(\mathbf{p_j} + \hat{\mathbf{x}}_\mathbf{i}) - g(\mathbf{p_{j-1}} + \hat{\mathbf{x}}_\mathbf{i}) \tag{10}$$

We now upper bound each term in this sum via the following claim.
Claim: For $1 \leq j \leq K$,

$$g(\mathbf{p_j} + \hat{\mathbf{x}}_\mathbf{i}) - g(\mathbf{p_{j-1}} + \hat{\mathbf{x}}_\mathbf{i}) \leq \frac{1}{\beta(g)}\left(g(\mathbf{x_i}_j \mathbf{e_j} + \hat{\mathbf{x}}_\mathbf{i}) - g(\hat{\mathbf{x}}_\mathbf{i})\right)$$

Proof of claim: Writing $\mathbf{p_j} = \mathbf{x_i}_j \mathbf{e_j} + \mathbf{p_{j-1}}$ and using $\mathbf{y}$ as a short hand for $\mathbf{p_{j-1}} + \hat{\mathbf{x}}_\mathbf{i}$, we can express the left hand side of the inequality in the claim as the telescoping sum

$$\sum_{\ell=1}^{\mathbf{x_i}_j} g(\ell \cdot \mathbf{e_j} + \mathbf{y}) - g((\ell - 1) \cdot \mathbf{e_j} + \mathbf{y})$$

Similarly, we can express the right hand side of the inequality in the claim as a telescoping sum

$$\frac{1}{\beta(g)} \sum_{\ell=1}^{\mathbf{x_i}_j} g(\ell \cdot \mathbf{e_j} + \hat{\mathbf{x}}_\mathbf{i}) - g((\ell - 1) \cdot \mathbf{e_j} + \hat{\mathbf{x}}_\mathbf{i}).$$

We now note that

$$\begin{aligned} & g(\ell \cdot \mathbf{e_j} + \mathbf{y}) - g((\ell - 1) \cdot \mathbf{e_j} + \mathbf{y}) \\ & \leq \frac{1}{\beta(g)} \left(g(\ell \cdot \mathbf{e_j} + \hat{\mathbf{x}}_\mathbf{i}) - g((\ell - 1) \cdot \mathbf{e_j} + \hat{\mathbf{x}}_\mathbf{i}) \right) \end{aligned}$$

This follows directly from the definition of $\beta(g)$. The claim follows. □

Combining (9) and (10) with the Claim, we get

$$\sum_{j=1}^{K} g(\mathbf{x_i}_j \mathbf{e_j} + \hat{\mathbf{x}}_\mathbf{i}) - g(\hat{\mathbf{x}}_\mathbf{i}) \geq \beta(g)(OPT - g(\hat{\mathbf{x}}_\mathbf{i})) \tag{11}$$

In each iteration $i + 1$, $0 \leq i < D$, the objective function is improved by a quantity Δ_{i+1}^{UG}, satisfying

$$\Delta_{i+1}^{UG} \geq \max_{1 \leq j \leq K} \frac{g(\mathbf{x_i}_j \mathbf{e_j} + \hat{\mathbf{x}}_\mathbf{i}) - g(\hat{\mathbf{x}}_\mathbf{i})}{\mathbf{x_i}_j}$$

Since the maximum on the right hand side of the above inequality is at least as large as the average gain in the objective function value per unit budget, we get

$$\Delta_{i+1}^{UG} \geq \frac{\sum_{j=1}^{K} g(\mathbf{x_i}_j \mathbf{e_j} + \hat{\mathbf{x}}_\mathbf{i}) - g(\hat{\mathbf{x}}_\mathbf{i})}{\sum_{j=1}^{K} \mathbf{x_i}_j}$$

Since $\sum_{j=1}^{K} \mathbf{x_i}_j \leq D$, we can combine the above inequality with (11) to get $\Delta_{i+1}^{UG} \geq \frac{\beta(g)}{D}(OPT - g(\hat{\mathbf{x}}_\mathbf{i}))$. □

Lemma A5.

$$OPT - \sum_{j=1}^{i} \Delta_j^{UG} \leq \prod_{j=1}^{i} \left(1 - \frac{\beta(g)}{D}\right) \cdot OPT$$

for $0 \leq i \leq D$

Proof: By induction. The base case with $i = 0$ is trivially true.

Assume the lemma holds for $i-1$ where $i \geq 1$. Then

$$
\begin{aligned}
& OPT - \sum_{j=1}^{i} \Delta_j^{UG} \\
& \leq OPT - \sum_{j=1}^{i-1} \Delta_j^{UG} - \frac{\beta(g)}{D}\left(OPT - \sum_{j=1}^{i-1} \Delta_j^{UG}\right) \\
& \qquad \text{[by Lemma A4]} \\
& = \left(1 - \frac{\beta(g)}{D}\right)\left(OPT - \sum_{j=1}^{i-1} \Delta_j^{UG}\right) \\
& \leq \left(1 - \frac{\beta(g)}{D}\right) \prod_{j=1}^{i-1} \left(1 - \frac{\beta(g)}{D}\right) \cdot OPT \\
& \qquad \text{[by inductive hypothesis]} \\
& = \prod_{j=1}^{i} \left(1 - \frac{\beta(g)}{D}\right) \cdot OPT
\end{aligned}
$$

□

Corollary A6.

$$\sum_{i=1}^{D} \Delta_i^{UG} \geq (1 - e^{-\beta(g)}) \cdot OPT$$

Proof: Let $\phi = \ln(1-x)$. Note that ϕ is concave and monotonically decreasing in the range $(0, 1]$. Then for $y_1, ..., y_D \in (0, 1]$, we know $\sum_{i=1}^{D} \frac{\phi(y_i)}{D} \leq \phi\left(\sum_{i=1}^{D} \frac{y_i}{D}\right)$ by Jensen's Inequality. Let $y_i \leftarrow \frac{\beta(g)}{D}$. Then

$$
\begin{aligned}
\frac{1}{D} \sum_{i=1}^{D} \ln\left(1 - \frac{\beta(g)}{D}\right) &\leq \ln\left(1 - \frac{\beta(g)}{D}\right) \\
\Rightarrow \sum_{i=1}^{D} \ln\left(1 - \frac{\beta(g)}{D}\right) &\leq \ln\left(1 - \frac{\beta(g)}{D}\right)^D \\
\Rightarrow \prod_{i=1}^{D} \left(1 - \frac{\beta(g)}{D}\right) &\leq \left(1 - \frac{\beta(g)}{D}\right)^D
\end{aligned}
$$

By Lemma A5 we have

$$
\begin{aligned}
OPT - \sum_{i=1}^{D} \Delta_i^{UG} &\leq \prod_{i=1}^{D} \left(1 - \frac{\beta(g)}{D}\right) \cdot OPT \\
&\leq \left(1 - \frac{\beta(g)}{D}\right)^D \cdot OPT \\
&\leq e^{-\beta(g)} \cdot OPT
\end{aligned}
$$

Then $\sum_{i=1}^{D} \Delta_i^{UG} \geq (1 - e^{-\beta(g)}) \cdot OPT$ □

Since, $g(\hat{\mathbf{x}}) = \sum_{i=1}^{D} \Delta_i^{UG}$, $g(\hat{\mathbf{x}} \geq (1 - e^{-\beta(g)}) \cdot OPT$, which completes the proof of Theorem 10. □

B.2 Heuristics

We provide pseudocode for Random and Population in Algorithm 2 and Algorithm 3, respectively.

Algorithm 2 Random $(\mathcal{M}, \mathbf{I})$

```
1: x̂ ← 0
2: while Σ_{k=1}^{K} X[k] ≤ D do
3:     k* ← random element from [K]
4:     x̂ ← x̂ + e_{k*}
5: end while
6:
7: return x̂
```

Algorithm 3 Population $(\mathcal{M}, \mathbf{I})$

```
1: x̂ ← 0
2: for k ∈ [K] do
3:     x̂_k ← Δ ⌊ n_k D / (Δ Σ_{i=1}^{K} n_i) ⌋
4: end for
5:
6: return x̂
```

C Experiments

C.1 Data

We incorporate population and mobility data into a disease model as a data-driven approach to simulate the effect of an individual's movement on disease spread and the impacts of an intervention.

FRED (A Framework for Reconstructing Epidemiological Dynamics) [3] boasts a high-detail agent-based dataset, which offers properties of each agent "as a separate individual each with her/his own unique social, familial, demographic, behavioral, and health characteristics". The data is organized by FIPS codes, where each individual residing within that code is assigned a unique ID number. Each ID number is associated with a school and/or workplace, household, age, etc. Each school/workplace has latitude/ longitude coordinates associated.

SafeGraph [4] is a data provider whose real-time datasets can be used to derive mobility patterns between different counties for a US state every week. It can also be used to create contact graphs that represent person-to-person interactions, observe movement trends, and understand clusters. It contains over 11M+ POI data and the data is tested for bias by comparing the panel data to the true proportions reported in the US Census. Pre-processing of the foot traffic data shows footfall at Points of Interest (POI). We obtain the representation of monthly mobility patterns among counties in Iowa and New Hampshire for the period of 1/1/18-1/31/18 (averaged to get daily representation). The graphs are denser than the mobility graphs derived from FRED and consist of directed edges denoting mobility to/from counties. One major disadvantage of SafeGraph is the fact that the data is very sparse for rural areas due to the low availability of strong mobile internet connections in those areas.

Safegraph provides some of its datasets for academic research on Dewey [5], which provides access to Monthly Patterns data [6]. This dataset contains monthly visitor and

demographic aggregations for points of interest (POIs) in the USA, derived from a panel of opted-in mobile devices. We obtained access to mobility patterns data for movements of people to different Points of Interest (POI) in the USA.

C.2 Mobility graph construction

1. FRED We derive a mobility graph representing daily commutes from the FRED data. We make the simplifying assumption that each individual will commute daily to their respective school/workplace, considering only commutes that cross into another subpopulation. We utilize this dataset to infer daily commute behavior by incrementing each edge weight for every individual who lives in one subpopulation and works in another. Each edge $e_{ij} \in E$ denotes a typical daily commute between counties i and j and the weight w_{ij} of each edge is the number of such individuals. The size n_k of each subpopulation k is simply taken as the number of individuals in the data for subpopulation k. Fig A in S1 Text illustrates the result of this construction.

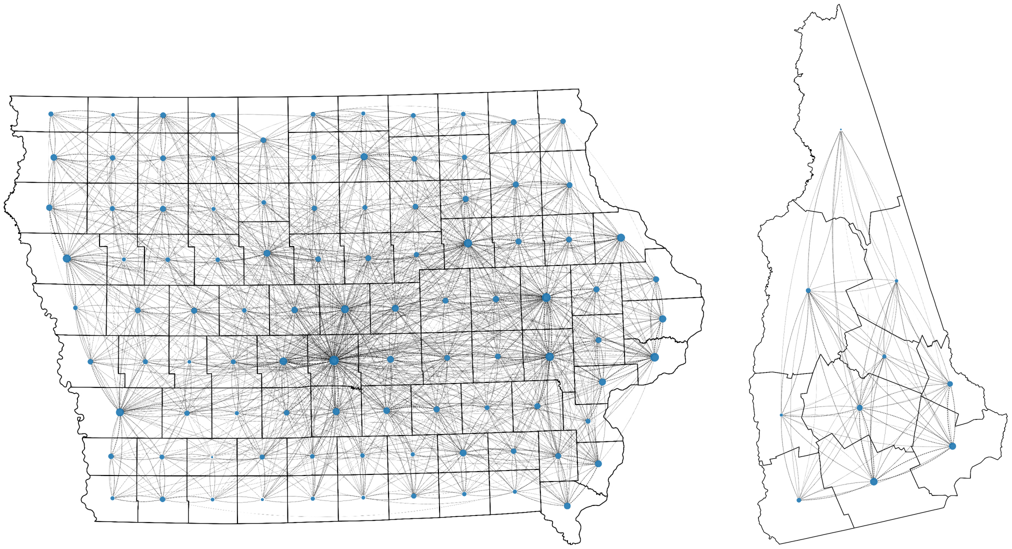

Fig A in S1 Text. Iowa (left) and New Hampshire (right) mobility graphs derived from FRED data. The size of each node is proportional to the population size of the subpopulation in which it is centered. Likewise, the width of each edge $e \in E_{ij}$ is proportional to its weight w_{ij} (number of individuals commuting from subpopulation i to subpopulation j). These figures were created using basemap [7] and U.S. Census Bureau Cartographic Boundary Files [8], which are not subject to copyright in the United States under 17 U.S.C. §105 [9] and are in the public domain.

2. SafeGraph We also derive a mobility graph similar to the mobility graph constructed from FRED data. As we have monthly data of visits to different POIs all over the USA with added information about the home location of each unique visitor, we construct a directed graph between communities with edges representing the movement of people from one community to another by aggregating the visits from home locations to the POIs. The aggregated mobility weights are then averaged to obtain approximate daily mobility values. Additionally, as SafeGraph provides additional information about POIs, with slight assumptions, we can also construct granular representations of the mobility graph by classifying visits to both workplace and non-workspace visits respectively.

State	Dataset	$|V|$	$|E|$	Density
**NH**	**FRED**	10	76	0.844
**NH**	**SafeGraph**	10	87	0.967
**IA**	**FRED**	90	1548	0.16
**IA**	**SafeGraph**	90	1660	0.171
**TX**	**FRED**	254	3872	0.06
**TX**	**SafeGraph**	254	31514	0.49

Table A in S1 Text. Comparison of FRED and SafeGraph mobility graph properties

C.3 Comparison to baselines

In addition to the experiments presented in the main text, we also evaluate the performance of our methods for a less infective value of λ on New Hampshire, Iowa, and Texas mobility graphs. For a lower λ value with a low budget, the greedy methods perform well compared to the baselines (Figs B in S1 Text, C in S1 Text, and D in S1 Text).

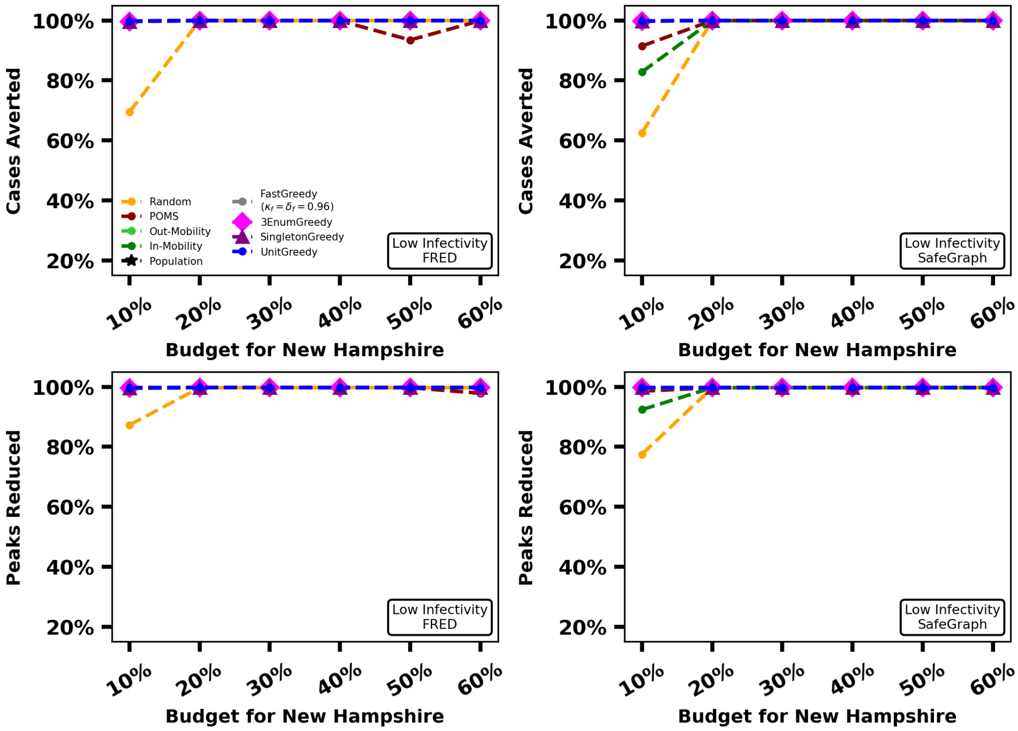

Fig B in S1 Text. Percentage TOTBURDEN and percentage MAXBURDEN reduced by all approaches for $\lambda = 0.347$ in New Hampshire for FRED (first column) and SafeGraph (second column).

C.4 Near-optimality of greedy algorithms

Here, we provide additional details regarding our method of indirectly establishing the near-optimality of larger problem instances (in this case, the state of Iowa). As in the

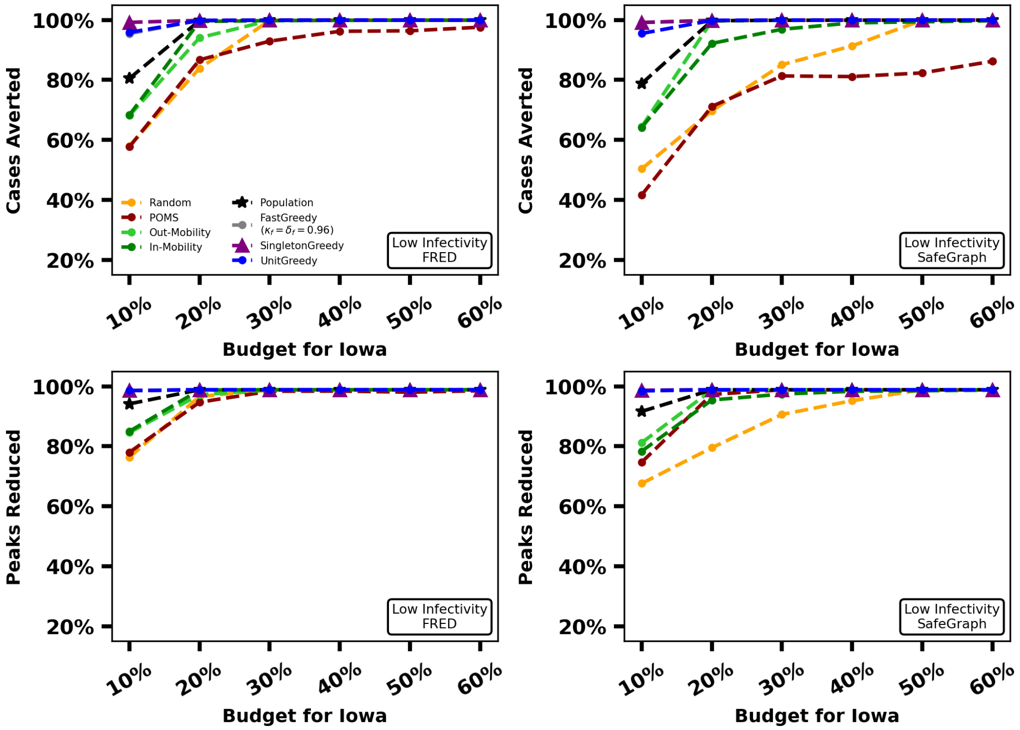

Fig C in S1 Text. Percentage TOTBURDEN and percentage MAXBURDEN reduced by UNITGREEDY, SINGLETONGREEDY, FASTGREEDY and baselines for $\lambda = 0.3475$ in Iowa for FRED (first column) and SafeGraph (second column).

main text, we present the following instance-specific DR-submodularity ratio definition.

Definition A7. *Let* $\hat{\mathbf{x}}_\mathbf{i}$ *denote the allocation after iteration* i *of* UNITGREEDY, *let* $\mathbf{x}^*$ *be an optimal solution, and let* $\mathbf{y}^* = \mathbf{0} \vee (\mathbf{x}^* - \hat{\mathbf{x}}_\mathbf{i})$

$$\beta(g, \hat{\mathbf{x}}_\mathbf{i}) := \frac{\sum_{j=1}^{K} g(\mathbf{y}_j^* \mathbf{e_j} + \hat{\mathbf{x}}_\mathbf{i}) - g(\hat{\mathbf{x}}_\mathbf{i})}{g(\mathbf{y}^* + \hat{\mathbf{x}}_\mathbf{i}) - g(\hat{\mathbf{x}}_\mathbf{i})} \tag{12}$$

Definition A7 comes from the proof of the claim in Lemma A4 (specifically, Eqs 9-11). Recall from the main text $g(\hat{\mathbf{x}}) \geq (1 - e^{-\beta(g,\hat{\mathbf{x}})}) \cdot OPT$. This is a Corollary to Lemma A4 - by combining the fact that $g(\mathbf{y}^* \mid \hat{\mathbf{x}}_\mathbf{i}) \leq OPT$ with Eq 11, we have

$$\sum_{j=1}^{K} g(\mathbf{y^*}_j \mathbf{e_j} + \hat{\mathbf{x}}_\mathbf{i}) - g(\hat{\mathbf{x}}_\mathbf{i}) \geq \beta(g)(g(\mathbf{y}^* + \hat{\mathbf{x}}_\mathbf{i}) - g(\hat{\mathbf{x}}_\mathbf{i})) \tag{13}$$

Then by definition A7, we get

$$\sum_{j=1}^{K} g(\mathbf{y^*}_j \mathbf{e_j} + \hat{\mathbf{x}}_\mathbf{i}) - g(\hat{\mathbf{x}}_\mathbf{i}) \geq \beta(g, \hat{\mathbf{x}})(g(\mathbf{y}^* + \hat{\mathbf{x}}_\mathbf{i}) - g(\hat{\mathbf{x}}_\mathbf{i})) \tag{14}$$

$g(\hat{\mathbf{x}}) \geq (1 - e^{-\beta(g,\hat{\mathbf{x}})}) \cdot OPT$ follows from the rest of the logic of the proof of Theorem 10.

Since we cannot calculate the optimal solution $\mathbf{x}^*$ directly (and $\beta(g, \hat{\mathbf{x}}_\mathbf{i})$ depends on $\mathbf{x}^*$), we cannot calculate $\beta(g, \hat{\mathbf{x}}_\mathbf{i})$ directly either. Instead, we estimate $\beta(g, \hat{\mathbf{x}}_\mathbf{i})$ with the following method.

First, let $\hat{\mathbf{x}}'_\mathbf{i}$ be a randomly sampled element from $\mathcal{K}(\hat{\mathbf{x}}_\mathbf{i})$ and let $S(\hat{\mathbf{x}}_\mathbf{i})$ be a set of such samples. $\mathcal{K}(\hat{\mathbf{x}}_\mathbf{i})$ denotes the k-swap neighborhood of $\hat{\mathbf{x}}_\mathbf{i}$, which is defined by the

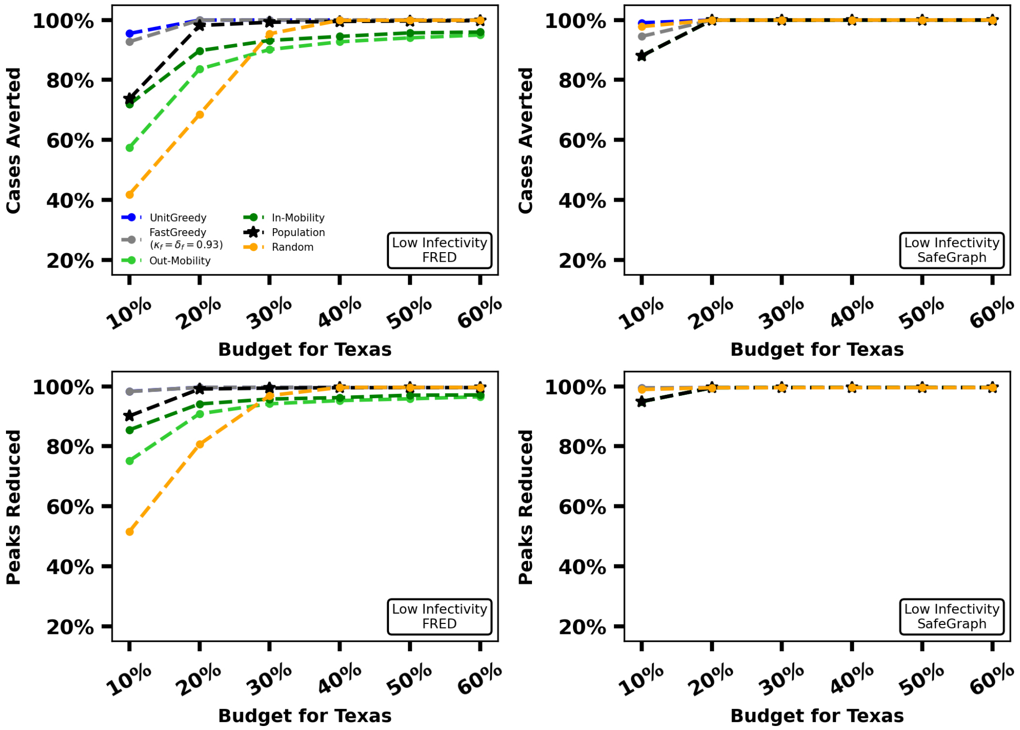

Fig D in S1 Text. Percentage TOTBURDEN and percentage MAXBURDEN reduced by UNITGREEDY, FASTGREEDY and baselines for $\lambda = 0.341$ in Texas for FRED (first column) and SafeGraph (second column).

allocations which can be reached by moving a bundle between two subpopulations k times. Let $\hat{\beta}'(g, \hat{\mathbf{x}}_\mathbf{i})$ denote an estimated value of $\beta(g, \hat{\mathbf{x}}_\mathbf{i})$ obtained from $\hat{\mathbf{x}}'_\mathbf{i} \in S(\hat{\mathbf{x}}_\mathbf{i})$.

Definition A8. *Let* $\hat{\mathbf{x}}_\mathbf{i}$ *denote the allocation after iteration* i *of* UNITGREEDY*, let* $\hat{\mathbf{x}}'_\mathbf{i} \in \mathcal{K}(\hat{\mathbf{x}}_\mathbf{i})$*, and let* $\mathbf{y}' = \mathbf{0} \vee (\hat{\mathbf{x}}'_\mathbf{i} - \hat{\mathbf{x}}_\mathbf{i})$

$$\hat{\beta}'(g, \hat{\mathbf{x}}_\mathbf{i}) := \frac{\sum_{j=1}^{K} g(\mathbf{y}'_j \mathbf{e}_\mathbf{j} + \hat{\mathbf{x}}_\mathbf{i}) - g(\hat{\mathbf{x}}_\mathbf{i})}{g(\mathbf{y}' + \hat{\mathbf{x}}_\mathbf{i}) - g(\hat{\mathbf{x}}_\mathbf{i})} \tag{15}$$

Let $\hat{\beta}'(g, S(\hat{\mathbf{x}}_\mathbf{i}))$ be the set of $\hat{\beta}'(g, \hat{\mathbf{x}}_\mathbf{i})$ values for each $\hat{\mathbf{x}}'_\mathbf{i} \in S(\hat{\mathbf{x}}_\mathbf{i})$. Let $\mathcal{I}$ be the set of iteration indices sampled from the course of a UNITGREEDY allocation. Then let $\hat{\beta}'(g, S(\hat{\mathbf{x}})) := \bigcup_{i \in \mathcal{I}} \hat{\beta}'(g, S(\hat{\mathbf{x}}_\mathbf{i}))$ and $\hat{\beta}(g, \hat{\mathbf{x}}) := \mathbb{E}[\hat{\beta}'(g, S(\hat{\mathbf{x}}))]$.

The resulting procedure we use to estimate $\beta(g, \hat{\mathbf{x}})$ is summarized below:

1. Determine intermediate UNITGREEDY allocation indices $\mathcal{I} = \{i_1, i_2, ..., i_{|\mathcal{I}|}\}$ to perturb. That is, select UNITGREEDY allocations $\hat{\mathbf{x}}_{\mathbf{i_1}}, \hat{\mathbf{x}}_{\mathbf{i_2}}, ..., \hat{\mathbf{x}}_{\mathbf{i}_{|\mathcal{I}|}}$ from which to construct $S(\hat{\mathbf{x}}_\mathbf{i})$. We chose 20 indices from which to sample, representing allocation budgets of $5\%, 10\%, ..., 100\%$.

2. Sample values from the k-swap neighborhood of $\hat{\mathbf{x}}_\mathbf{i}$ for each $i \in \mathcal{I}$. For our experiment, we selected $k = 5$ for New Hampshire and $k = 10$ for Iowa. We sampled 400 values from $\mathcal{K}(\hat{\mathbf{x}}_\mathbf{i})$ for each $i \in \mathcal{I}$ (a total of 8000 samples for each $\hat{\mathbf{x}}$).

3. Calculate $\hat{\beta}(g, \hat{\mathbf{x}})$ using $\hat{\beta}'(g, \hat{\mathbf{x}}_\mathbf{i})$ for each $i \in \mathcal{I}$ and each $\hat{\mathbf{x}}'_\mathbf{i} \in S(\hat{\mathbf{x}}_\mathbf{i})$.

Due to the similarity of Lemmas A1 and A4, we can construct $\alpha(g, \hat{\mathbf{x}}) \geq \alpha(g)$ and find an estimate $\hat{\alpha}(g, \hat{\mathbf{x}})$ in a similar manner for allocations chosen by ℓ-ENUMGREEDY, and SINGLETONGREEDY. We remark that we do not currently have any guarantees with respect to the number of samples required for confidence bounds of $\hat{\beta}(g, \hat{\mathbf{x}})$.

C.5 Reproducibility

We include our experimental framework which contains code for data pre-processing, metapopulation disease spread model, vaccine allocation, and data processing. The specific steps are located in a "README" file included with the code.

Parameters We fix δ and η to be 0.25 and 0.2786, respectively, taken from [10]. We selected values of λ at approximately 0.347 and 0.535 to result in 20% and 70% for each population becoming infected without vaccination, respectively. We explored a wide range of λ values to reflect from 10% up to 90% of a population infected. All simulations are run for up to $T = 1500$ timesteps, or until infections die out. In all experiments, we define the number of vaccines in a bundle to be 0.03% of the total population size. For Random and POMS, we evaluate the average of 20 and 5 runs, respectively. The experiments were carried out on 4 servers whose specifications are given in Table 2.

System	CPUs	Memory	Storage
AMD EPYC 7763 64-Core Processor	128	2.10 TB	28 TB
AMD EPYC 7763 64-Core Processor	128	2.11 TB	56 TB
AMD EPYC 7763 64-Core Processor	128	1.05 TB	28 TB
Intel Xeon E5-2683 v4 2.10 GHz	64	0.53 TB	59 TB

Table B in S1 Text. System Specifications

D Related works

Vaccine Allocation: Recent effects of the COVID-19 pandemic on society have garnered a lot of interest in the topic of effective vaccine allocation methods. This has led to different approaches to optimize this problem. On that note, one of the previous works assumes vaccination as a cost and proposes the convex optimization of that cost function in a network model [11], while another uses probabilistic methods to characterize the optimal distribution of vaccines in a given contact network [12]. On the other hand, MIN-CONN [13] provides a heuristic for effective vaccine allocation while DAVA [14, 15] and its variants pose the problem as a combinatorial optimization and propose polynomial time heuristics to solve it. More recent works include analysis of the effects of the dosage pattern of vaccines [16], reinforcement learning approaches [17, 18], and vaccine allocation in metapopulation models [19, 20].

Submodularity: Various prior works exist in submodularity with traditional works focusing on the analysis of greedy and local improvement heuristics for the matroid optimization problem [21], its applications [22], and submodular function minimization [23] and maximization [24] under different scenarios. Some previous work also explores the connection between submodularity and convexity and find a polynomial-time algorithm to find the minimum of a submodular set-function [25]. Similarly, a line of prior works also explores lattice submodularity [1, 26] and minimization [27]. On the other hand, another direction of research has aimed at PAC-style learning of submodular functions in a distributional setting [28] and using submodularity on online resource allocation problems [29].

General Submodular Functions and Variants: While prior works exploring submodularity addressed submodular function minimization with a lower bound [30, 31], proper selection of strong submodular observation sets [32], and polynomial-time

submodular function approximation [33], some prior works have also explored decomposable submodular function minimization problems [34], maximization of non-monotone submodular functions [35], and minimization of symmetric submodular functions [36]. Similarly, other works focus on the application of submodular function maximization with routing constraints in real-life networks [37], epidemiological constraints [38, 39], combinatorial strongly polynomial algorithms for submodular function minimization [40] and stochastic functions that are given as an expectation of functions over a distribution by directly optimizing a combination of bias and variance [41]. More recently, there has been work on non-submodular function optimization, which can be used to derive approximation guarantees through a function's distance to submodularity, known as the submodularity ratio [1, 42, 43].

E Notation summary

Variable	Definition
$\mathcal{M}$	Metapopulation disease model instance
K, T	Number of subpopulations, size of time window
r_i	Population density correlated λ-multiplier for subpopulation i
n_i	Size of subpopulation i
$\mathbf{W}, w_{ij}$	Mobility matrix, mobility from subpopulations i to j
q_i^t	Force of infection in subpopulation i at time t
$\mathbf{I}$	Initial infection vector
$\lambda, 1/\eta, 1/\delta$	Infectivity, latency period, infectious period
$S_i^t, E_i^t, I_i^t, R_i^t$	Number of individuals which are Susceptible, Exposed, Infected, and Recovered (respectively) in subpopulation i at time t
$f(\mathbf{x} \mid \mathcal{M}, \mathbf{I})$	Measure of disease-spread conditioned on model and initial infections
$g(\mathbf{x} \mid \mathcal{M}, \mathbf{I})$	Measure of reduction in disease-spread conditioned on model and initial infections

Table C in S1 Text. Metapopulation model notation.

Variable	Definition
D	Vaccine budget
$\mathbf{x}$	Vaccine allocation solution vector
$\beta(g)$	DR-submodularity ratio
$\alpha(g)$	Submodularity ratio
τ_f	FastGreedy threshold
$\mathbf{x}$	Vaccine allocation solution vector
β_f	FastGreedy DR-submodularity ratio
κ_f	FastGreedy Threshold decrease rate
δ_f	FastGreedy DR-submodularity ratio decrease rate

Table D in S1 Text. Problem formulations and algorithm notation.

References

1. Qian C, Zhang Y, Tang K, Yao X. On Multiset Selection With Size Constraints. Proceedings of the AAAI Conference on Artificial Intelligence. 2018;32(1).

2. Alon N, Gamzu I, Tennenholtz M. Optimizing budget allocation among channels and influencers. In: Mille A, Gandon F, Misselis J, Rabinovich M, Staab S, editors. Proceedings of the 21st World Wide Web Conference 2012, WWW 2012, Lyon, France, April 16-20, 2012. ACM; 2012. p. 381–388. Available from: https://doi.org/10.1145/2187836.2187888.

3. Grefenstette J, Brown S, Rosenfeld R, Depasse J, Stone N, Cooley P, et al. FRED (A Framework for Reconstructing Epidemic Dynamics): An Open-Source Software System for Modeling Infectious Diseases and Control Strategies Using Census-Based Populations. BMC Public Health. 2013;13(1):940.

4. SafeGraph. Places Data Curated for Accurate Geospatial Analytics; 2023. https://safegraph.com.

5. Dewey. Academic Research Data; 2022. https://www.deweydata.io/.

6. SafeGraph. Patterns; 2023. https://docs.safegraph.com/docs/monthly-patterns.

7. Matplotlib Development Team. Basemap Toolkit Documentation; 2025. https://matplotlib.org/basemap/stable/.

8. U S Census Bureau. Cartographic Boundary Files; 2025. https://www.census.gov/geographies/mapping-files/time-series/geo/carto-boundary-file.html.

9. 17 U.S.C. § 105 - Subject matter of copyright: United States Government works; 2024. United States Code. Available from: https://www.govregs.com/uscode/title17_chapter1_section105.

10. Sen P, Kandula S, Shaman J. Differential Effects of Intervention Timing on COVID-19 Spread in the United States. Science Advances. 2020;6(49).

11. Medlock J, Galvani A. Optimizing Influenza Vaccine Distribution. Science. 2009;325(5948):1705–8.

12. Borgs C, Chayes J, Ganesh A, Saberi A. How to distribute antidote to control epidemics. Random Structures & Algorithms. 2010;37(2):204–222.

13. Prakash BA, Adamic L, Iwashyna T, Tong H, Faloutsos C. Fractional immunization in networks. In: Proceedings of the 2013 SIAM international conference on data mining. SIAM; 2013. p. 659–667.

14. Zhang Y, Prakash BA. Scalable vaccine distribution in large graphs given uncertain data. In: Proceedings of the 23rd ACM International Conference on Conference on Information and Knowledge Management; 2014. p. 1719–1728.

15. Zhang Y, Prakash BA. Data-aware vaccine allocation over large networks. ACM Transactions on Knowledge Discovery from Data (TKDD). 2015;10(2):1–32.

16. Matrajt L, Eaton J, Leung T, Dimitrov D, Schiffer JT, Swan DA, et al. Optimizing vaccine allocation for COVID-19 vaccines shows the potential role of single-dose vaccination. Nature communications. 2021;12(1):3449.

17. Hao Q, Huang W, Xu F, Tang K, Li Y. Reinforcement Learning Enhances the Experts: Large-scale COVID-19 Vaccine Allocation with Multi-factor Contact Network. In: Proceedings of the 28th ACM SIGKDD Conference on Knowledge Discovery and Data Mining; 2022. p. 4684–4694.

18. Rey D, Hammad AW, Saberi M. Vaccine allocation policy optimization and budget sharing mechanism using reinforcement learning. Omega. 2023;115:102783.

19. Venkatramanan S, Chen J, Gupta S, Lewis B, Marathe M, Mortveit H, et al. Spatio-temporal optimization of seasonal vaccination using a metapopulation model of influenza. In: 2017 IEEE International Conference on Healthcare Informatics (ICHI). IEEE; 2017. p. 134–143.

20. Ye Y, Zhang Q, Wei X, Cao Z, Yuan HY, Zeng DD. Equitable access to COVID-19 vaccines makes a life-saving difference to all countries. Nature human behaviour. 2022;6(2):207–216.

21. Fisher ML, Nemhauser GL, Wolsey LA. An analysis of approximations for maximizing submodular set functions—II. Springer; 1978.

22. Edmonds J. Submodular functions, matroids, and certain polyhedra. In: Combinatorial Optimization—Eureka, You Shrink! Papers Dedicated to Jack Edmonds 5th International Workshop Aussois, France, March 5–9, 2001 Revised Papers. Springer; 2003. p. 11–26.

23. Iwata S. Submodular function minimization. Mathematical Programming. 2008;112:45–64.

24. Krause A, Golovin D. Submodular function maximization. Tractability. 2014;3(71-104):3.

25. Lovász L. Submodular functions and convexity. Mathematical Programming The State of the Art: Bonn 1982. 1983;.

26. Soma T, Kakimura N, Inaba K, Kawarabayashi K. Optimal Budget Allocation: Theoretical Guarantee and Efficient Algorithm. In: Proceedings of the 31st International Conference on Machine Learning. PMLR; 2014. p. 351–59.

27. Topkis DM. Minimizing a submodular function on a lattice. Operations research. 1978;26(2):305–321.

28. Balcan MF, Harvey NJ. Learning submodular functions. In: Proceedings of the forty-third annual ACM symposium on Theory of computing; 2011. p. 793–802.

29. Streeter M, Golovin D. An online algorithm for maximizing submodular functions. Advances in Neural Information Processing Systems. 2008;21.

30. Iyer RK, Bilmes JA. Submodular optimization with submodular cover and submodular knapsack constraints. Advances in neural information processing systems. 2013;26.

31. Svitkina Z, Fleischer L. Submodular approximation: Sampling-based algorithms and lower bounds. SIAM Journal on Computing. 2011;40(6):1715–1737.

32. Krause A, McMahan HB, Guestrin C, Gupta A. Robust Submodular Observation Selection. Journal of Machine Learning Research. 2008;9(12).

33. Goemans MX, Harvey NJA, Iwata S, Mirrokni V. In: Approximating Submodular Functions Everywhere; 2009. p. 535–544.

34. Stobbe P, Krause A. Efficient minimization of decomposable submodular functions. Advances in Neural Information Processing Systems. 2010;23.

35. Feige U, Mirrokni VS, Vondrák J. Maximizing non-monotone submodular functions. SIAM Journal on Computing. 2011;40(4):1133–1153.

36. Queyranne M. Minimizing symmetric submodular functions. Mathematical programming. 1998;82:3–12.

37. Zhang H, Vorobeychik Y. Submodular optimization with routing constraints. In: Proceedings of the AAAI conference on artificial intelligence. vol. 30; 2016.

38. Adhikari B, Lewis B, Vullikanti A, Jiménez JM, Prakash BA. Fast and near-optimal monitoring for healthcare acquired infection outbreaks. PLoS computational biology. 2019;15(9):e1007284.

39. Jang H, Fu A, Cui J, Kamruzzaman M, Prakash BA, Vullikanti A, et al. Detecting Sources of Healthcare Associated Infections. Proceedings of the AAAI Conference on Artificial Intelligence. 2023;(4):4347–4355.

40. Iwata S, Fleischer L, Fujishige S. A combinatorial strongly polynomial algorithm for minimizing submodular functions. Journal of the ACM (JACM). 2001;48(4):761–777.

41. Staib M, Wilder B, Jegelka S. Distributionally robust submodular maximization. In: The 22nd International Conference on Artificial Intelligence and Statistics. PMLR; 2019. p. 506–516.

42. Das A, Kempe D. Approximate Submodularity and Its Applications: Subset Selection, Sparse Approximation and Dictionary Selection. Journal of Machine Learning Research. 2018;19(3):1–34.

43. Kuhnle A, Smith D, Crawford V, Thai M. Fast Maximization of Non-Submodular, Monotonic Functions on the Integer Lattice. In: Proceedings of the 35th International Conference on Machine Learning; 2018. p. 2786–95.
